# Know your limits; miniCOI metabarcoding fails with key marine zooplankton taxa

**DOI:** 10.1093/plankt/fbae057

**Published:** 2024-11-02

**Authors:** Aitor Albaina, Rade Garić, Lidia Yebra

**Affiliations:** Department of Zoology and Animal Cell Biology, Faculty of Science and Technology, Universidad del País Vasco/Euskal Herriko Unibertsitatea (UPV/EHU), Barrio Sarriena s/n, 48940 Leioa, Bizkaia, Spain; Institute for Marine and Coastal Research, University of Dubrovnik, Kneza Damjana Jude 12, 20000 Dubrovnik, Croatia; Centro Oceanográfico de Málaga (IEO, CSIC), Explanada de San Andrés (Muelle 9), Puerto de Málaga, 29002 Málaga,Spain

**Keywords:** appendicularia, DNA metabarcoding, miniCOI, *Oithona similis*, zooplankton biodiversity

## Abstract

Eleven years after the publication of the first work applying deoxyribonucleic acid (DNA) metabarcoding to zooplankton communities, the commonly known “miniCOI” barcode is widely used, becoming the marker of choice. However, several primer combinations co-exist for this barcode and a critical evaluation of their performance is needed. This article reviews the misperformance of miniCOI metabarcoding with marine zooplankton communities, comparing them to microscopy and/or other universal markers. In total, misperformances were reported for 26 zooplankton taxa, including 18 copepods and five tunicates. We report a detection failure with Class Appendicularia and contrasting performances for *Oithona similis* (from good correspondence to detection failure), two worldwide abundant taxa with a crucial role in the marine pelagic realm. A combination of forward primer mismatches, the presence of long poly-T inserts and a low number of reference sequences would explain the failure to detect appendicularians. However, the contrasting performance with *O. similis* would correspond to distinct numbers of mismatches in the forward primer in different lineages within this cryptic taxon. This is reinforced by the report of similar patterns with other locally abundant zooplankton taxa. Therefore, we strongly call for the use of miniCOI in combination with alternative methods capable of addressing these limitations.

## INTRODUCTION

Before the democratization of massive parallel sequencing (MPS), the biodiversity assessment of whole planktonic communities with deoxyribonucleic acid (DNA) sequencing was rare and based on Sanger sequencing preceded by either individual taxa sorting or a cloning step (first studies by Bucklin *et al.*, 2010 and Machida *et al.*, 2009, respectively). Both methods are low-throughput with a very limited capacity to disclose the huge diversity encompassed in planktonic biocenoses. However, since the pioneer work of Lindeque *et al.* (2013), DNA metabarcoding has become the preferred alternative, allowing a high taxonomic resolution and unrivaled high-throughput capacities, with a sensitivity comparable to that of quantitative polymerase chain reaction (qPCR) species-specific assays (e.g. Santoferrara, 2019; Laakmann *et al.*, 2020; Keck *et al.*, 2022).

**Fig. 1:**
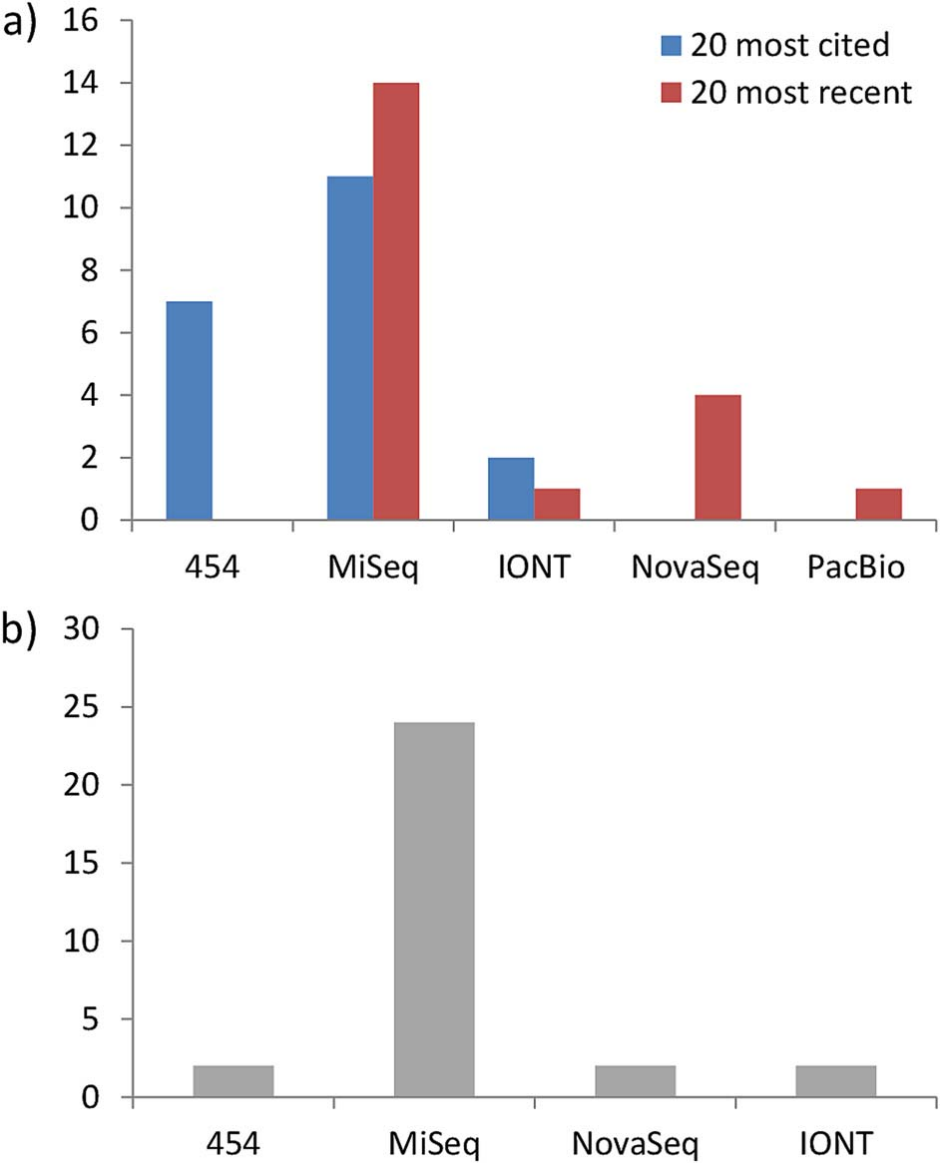
Different sequencing methods used in the literature. (**a**) Results of searching articles on marine metazoan zooplankton metabarcoding or metagenetics (20 most cited and 20 most recent articles); (**b**) results of previous search including CO1, COI or COX1, and comparison vs. microscopy and/or other universal marker (30 articles reviewed for the analysis of miniCOI metabarcoding performance). See Fig. S1 for literature search details. References’ details in Tables S1, S2 and S3.

**Fig. 2:**
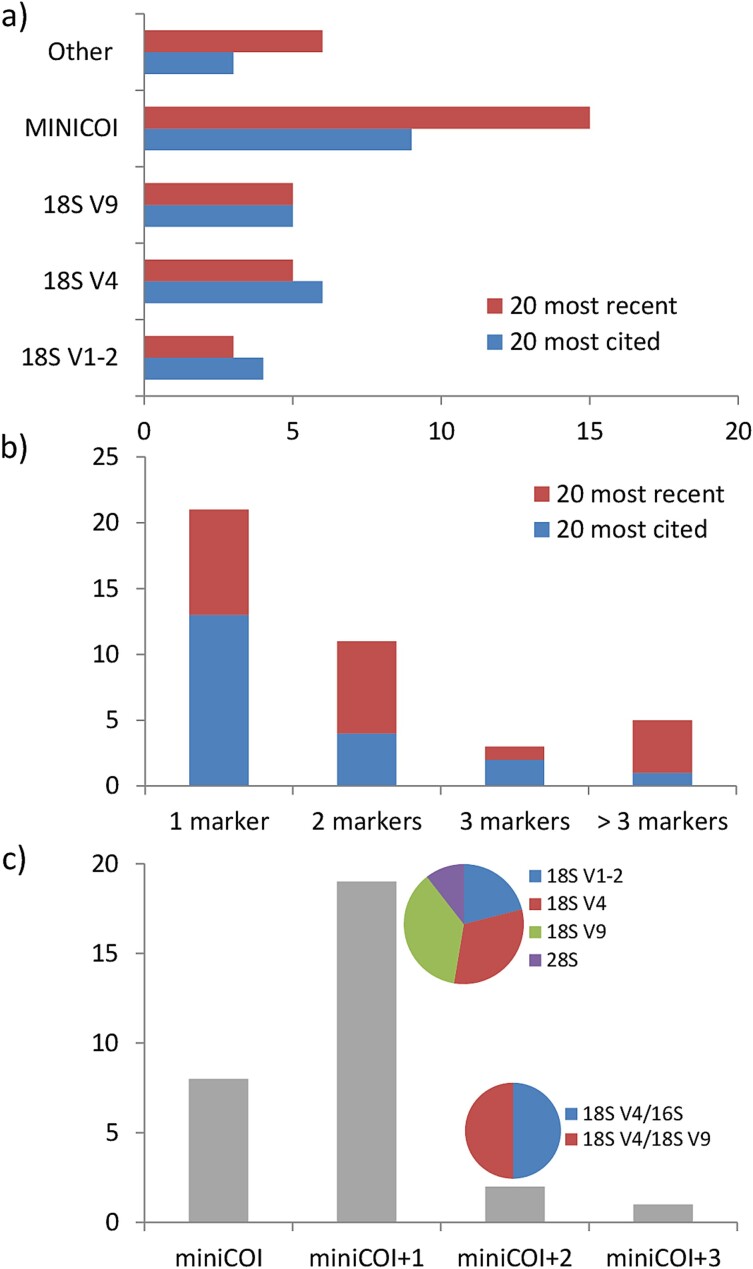
Different barcodes used in the literature. (**a**) Different markers used in the 20 most cited and 20 most recent reviewed articles; (**b**) number of markers used per article (20 most cited and 20 most recent); (**c**) markers used in combination with miniCOI in the 30 articles reviewed for the analysis of miniCOI metabarcoding performance (note that Zhang *et al.*, 2018 combined three different COI primers along with the 18S V4 one). See Fig. S1 for literature search details. References’ details in Tables S1, S2 and S3. Further details in Table S4.

As of 19 November 2023, Web of Science (WoS) yielded 199 article-type results when searching for [(TS = (zooplankton)) AND (TS = (metabarcoding OR metagenetics))] (Fig. S1. TS = Topic in WoS; full details at https://images.webofknowledge.com/images/help/WOS/hs_advanced_fieldtags.html). Trends for the last decade of marine metazoan zooplankton metabarcoding arise when we focus on the 20 most cited (Table S1) and also the 20 most recently published papers (Table S2) that assessed either field and/or mock samples. While the former set included papers ranging from 2013 to 2020, the latter did for the period 2022–2023 (Tables S1 and S2). Although first tries relied on the Roche’s 454 technology, the modern approach usually involves Illumina’s sequencing machines (Fig. 1a). The latter offering a higher value for money, albeit at a reduced sequence length (although nowadays approaching that of the 454 machine). In the first successful attempts to apply DNA metabarcoding to marine metazoan zooplankton communities, more than half of the published works applied a single barcode. Nowadays, however, most of the studies combine two or more barcodes trying to maximize the ranges of taxa detected (Fig. 2b).

### MiniCOI as marker of choice

Regarding the barcode to be sequenced, the golden standard region of DNA barcoding, the mitochondrial cytochrome oxidase I (COI or CO1 or COX1; COI hereinafter) region (Folmer *et al.*, 1994), has progressively become the marker of choice for marine metazoan zooplankton (Fig. 2a). COI is the most used and recommended amplicon for metazoans mainly due to its high taxonomic resolution and the deepest reference sequence databases (e.g. Andújar *et al.*, 2018). It is also the best-covered barcode in zooplankton databases (Bucklin *et al.*, 2021).

Due to the sequence length limits of most common MPS machines, Folmer’s original barcode (~650 bp; Folmer *et al.*, 1994) had to be restricted to approximately half of its length. For this reason, COI barcodes in metabarcoding encompass the anterior or the posterior half region, being commonly known as “miniCOI” barcodes (the most used one having ~ 313 bp, Leray *et al.*, 2013). Nowadays, miniCOI barcodes have become the method of choice for zooplankton metabarcoding (Fig. 2a). While the taxonomic resolution capacity of miniCOI with zooplankton seems to be comparable to that of the original Folmer amplicon (e.g. Ershova *et al.*, 2021), primer binding sites are not highly conserved (Leray *et al.*, 2013; Deagle *et al.*, 2014). Thus, making them prone to present mismatches with a wider range of taxa than other short universal markers, such as the 18S ribosomal RNA (rRNA) V1–2, V4 or V9 regions. Because of this, the miniCOI region is expected to be more biased regarding the amplification of certain taxa (e.g. Laakmann *et al.*, 2020). However, to date, only the “positive results” of this technique have been reviewed, as with the enhanced species richness assessment or the (semi-)quantitative nature for certain taxa reported in several works (Santoferrara, 2019; Keck *et al.*, 2022). In this regard, and to the best of our knowledge, this is the first review of the “negative results” (= hereinafter misperformance) of miniCOI DNA metabarcoding on marine zooplankton communities.

**Fig. 3:**
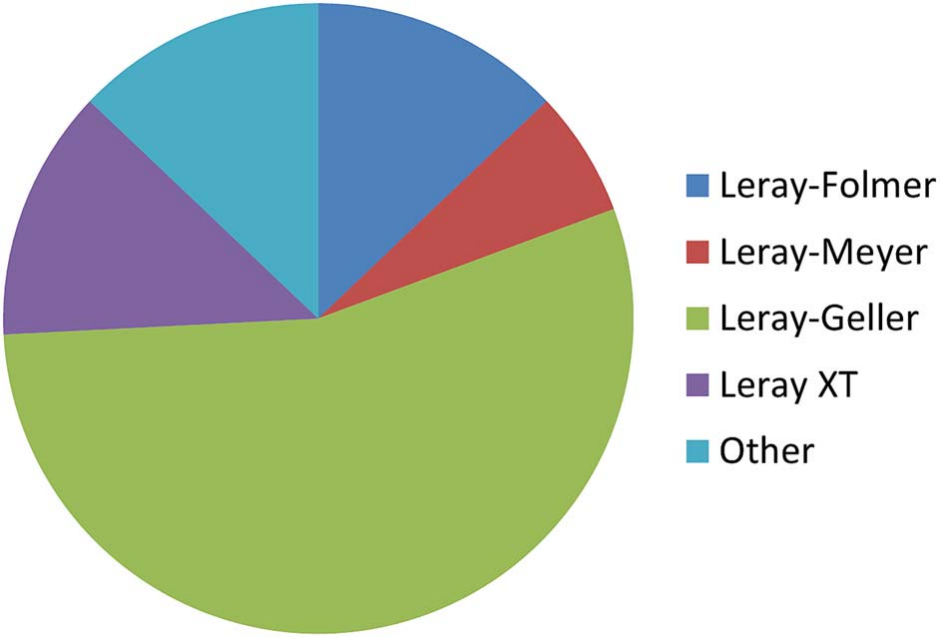
Different miniCOI primers combinations used in the 30 articles reviewed for the analysis of miniCOI metabarcoding performance. See Fig. S1 for literature search details. References’ details in Table S3.

Within the current framework of an increasing lack of plankton taxonomists, critically addressing the limitations of alternative identification methods is pivotal and urgent. Without proper comparison and acknowledgement of the differences between molecular and morphological identifications, we could be missing important information of the field communities’ composition without noticing it, even with locally abundant taxa. Moreover, while the forward and reverse primer combination for the most common barcodes accompanying the miniCOI (notably 18S rRNA gene regions V1–2, V4 or V9, Fig. 2c) has hardly been modified and there is a large consensus on them (Fonseca *et al.*, 2010; Zhan *et al.*, 2013 and either Amaral-Zettler *et al.*, 2009 or http://www.earthmicrobiome.org/emp-standard-protocols/18s/, respectively), the same does not apply to the miniCOI barcode/s (Fig. 3). The most used miniCOI primer combination pairs are the Leray’s mlCOIintF forward primer (Leray *et al.*, 2013) with a reverse primer ranging from (i) the Folmer’s HCO2198 (with no ambiguities; Folmer *et al.*, 1994), (ii) to the Meyer’s dgHCO2198 (with two ambiguous nucleotides; Meyer, 2003) and (iii) the Geller’s jgHCO2198 (with a total of eight ambiguities, corresponding to every third codon position, and including also inosines; Geller *et al.*, 2013; Table I). More recently, Wangensteen *et al.* (2018) added extra ambiguities and also inosines in the Leray mlCOIintF forward primer that combined with the original Geller reverse primer formed the so-called “Leray XT” primers (Table I). The degenerated base inosine (I) has traditionally been included to increase markers’ universality as it has affinity to all four DNA nucleotides, but with the following order of stability: I-C > I-A > I-T ≈ I-G (Martin *et al.*, 1985). In this review, we have followed a conservative approach when analyzing mismatches in the primer region and have considered inosines as universal nucleotides potentially matching all bases (as in Piñol *et al.*, 2019).

**Table I:** MiniCOI primers.

Direction	Primer	Sequence	Reference	Combination
Forward	mlCOIintF	GG**W**AC**W**GG**W**TGAAC**W**GT**W**TA**Y**CC**Y**CC	Leray *et al.*, 2013	
Reverse	HCO2198	TAAACTTCAGGGTGACCAAAAAATCA	Folmer *et al.*, 1994	Leray–Folmer
	dgHCO2198	TAAACTTCAGGGTGACCAAA**R**AA**Y**CA	Meyer, 2003	Leray–Meyer
	jgHCO2198	TA**I**AC**Y**TC**I**GG**R**TG**I**CC**R**AA**R**AA**Y**CA	Geller *et al.*, 2013	Leray–Geller
Forward	mlCOIintF-XT	GG**W**AC**WR**G**W**TG**R**AC**WI**T**I**TA**Y**CC**Y**CC	Wangensteen *et al.*, 2018	
Reverse	jgHCO2198	TA**I**AC**Y**TC**I**GG**R**TG**I**CC**R**AA**R**AA**Y**CA	Geller *et al.*, 2013	Leray XT

Most common miniCOI primers (all showed in 5’to 3’direction) and their combinations’ nomenclature; ambiguous nucleotides (W = A/T; Y=C/T; R = A/G) and inosines (I, considered as universal nucleotide, see text for further details) in bold.

### Metasynthesis of studies comparing miniCOI metabarcoding with microscopy or other universal marker

The present work evaluates the limitations of the distinct miniCOI primer combinations for assessing marine zooplankton biodiversity applying a metasynthesis approach (i.e. the systematic review and integration of findings from qualitative studies). We focus on reported misperformances against alternative methods (either microscopy and/or other universal markers). With this aim, we added the term [AND (TS = (CO1 OR COI OR COX1))] to the previous WoS search and reviewed the 63 articles yielded. We combined this broad systematic review with the addition of related works that did not appear in it, in order to get a broader picture of the reviewed field (Fig. S1). Thus, 11 works were added to the previous set due to (i) either being published earlier than 19 of November 2023 but yet not included in the WoS repository (Feng *et al.*, 2023; Ohnesorge *et al.*, 2023) or (ii) that did not fit the searched terms but did report data comparing miniCOI metabarcoding against alternative taxonomy methods (nine, including five that made it to the final dataset: Deagle *et al.*, 2018; Harvey *et al.*, 2017, 2018; Matthews and Blanco-Bercial, 2023; Matthews and Ohman, 2023; Fig. S1). Among these 74 works, we looked for papers that performed a parallel assessment by microscopy and/or other universal marker (as to compare with), with a focus on marine metazoan zooplankton communities including also holoplankton taxa (Fig. S1). This resulted in 30 articles that represent the core of this review (Table S3). The majority of the studies (24 out of 30) employed the MiSeq sequencing platform (Fig. 1b), while the remaining works utilized the 454 (2), NovaSeq (2), or Ion Torrent (2) platforms. From them, 22 applied at least a second marker (Fig. 2c), 20 identified taxa under the stereomicroscope and 12 did both (Table S4). Next, we revised their main text, figures and tables, as well as supplementary materials, when available. We quoted any reference of the authors to biases in detection of zooplankton taxa focusing on both (i) detection failures (false negatives) and (ii) pronounced biases reported for locally abundant taxa (i.e. more than an order of magnitude lower in relative frequencies when compared to an alternative method; see Table S4 for further details). By focusing on locally abundant taxa, we aimed to minimize the influence of confounding factors such as stochastic effects due to low abundances and/or specific bioinformatics thresholds for a positive detection (McLaren *et al.*, 2019; Santoferrara, 2019; Drake *et al.*, 2022). The reviewed articles were published from 2017 to 2023, being most of them (19 out of 30) published in the period 2021–2023, reducing somehow the putative bias coming from the scarcity of specific reference sequences before the advent of comprehensive zooplankton DNA databases (e.g. Bucklin *et al.*, 2021). We observed that the most frequently used database was National Center for Biotechnology Information (NCBI; GenBank), followed by MetaZooGene (MZG, Bucklin *et al.*, 2021; https://metazoogene.org/) (Fig. S2). From the reviewed works, seven also included locally generated Sanger sequences, and five articles combined NCBI with one or two publicly available databases (Table S4). Unfortunately, one of the articles (Novotny *et al.*, 2022) did not specify the database used.

### Primer mismatch analysis

Primer mismatches are considered the main factor contributing to amplification bias in DNA metabarcoding (Elbrecht and Leese, 2015, 2017; Piñol *et al.*, 2015, 2019). This issue is particularly critical for the relatively poorly conserved primer regions of the miniCOI marker. To mitigate this, an increasing number of ambiguities have been introduced (Table I).

Currently, the extensive coverage of curated DNA databases (e.g. GenBank, MIDORI, BOLD and the zooplankton-specific MZG) enables the quantification of primer mismatches in taxa with available reference sequences. Although primer mismatch analysis is already used in the design of metabarcoding markers (e.g. Elbrecht and Leese, 2017), the present study represents, to the best of our knowledge, the first application to characterize PCR amplification biases in zooplankton taxa. For this purpose, available COI sequences spanning the primer regions are aligned, and the nature and location of any mismatches are recorded.

It is well-known that mismatches located in the 3′ end region of a primer have significantly larger effects on priming efficiency than more 5′ located mismatches (e.g. Christopherson *et al.*, 1997; Stadhouders *et al.*, 2010). However, the effect of mismatches located more internally (“internal mismatches” hereinafter) have been less assayed (e.g. Sipos *et al.*, 2007; Piñol *et al.*, 2019). According to Christopherson *et al.* (1997), five to six internal mismatches would correspond to between 13 to 22-fold and 79 to 108-fold fewer amplifications, respectively. More recently, simulations by Lefever *et al.* (2013) suggested that four or more internal mismatches would prevent amplification in most of the cases. Moreover, DNA metabarcoding, where hundreds/thousands of target species compete for primers’ affinity, represents a stringer media where a certain number of mismatches in the primer region might be associated with a higher bias than the modeled/tested in simpler samples (e.g. Sipos *et al.*, 2007; McLaren *et al.*, 2019). In this regard, Piñol *et al.* (2015) showed that primer mismatches alone would explain 75% of amplification success in DNA metabarcoding when applying a different miniCOI barcode to a set of mock samples composed of arthropods. According to these authors, just one/two internal mismatches would correspond to a 1/10 misdetection, while for three or more mismatches (with one of them in the last three nucleotides from the 3’end) would be over 1/1000.

## METASYNTHESIS OF MINICOI PERFORMANCE

The literature reviewed included 13 papers that studied zooplankton from the Atlantic Ocean and 11 that focused on the Pacific Ocean; 2 studies provided samples from each of the remaining ocean basins (i.e. Indian, Arctic and Southern; Table S4). Among them, 16 papers reported biases with the class Copepoda and 19 reported biases with other zooplankton groups (Table II). The Leray–Geller primer combination was the most used (17 out of 30), followed by Leray XT with 4 works (Table III).

**Table II:** MiniCOI performance review synthesis-I.

Taxon	Studies showing misperformance (codes in Table S3)						
Copepods	AO	PO	IO	ArO	SO	Undet.	Total
*Oithona similis*	8,25,29	11,14,15,18,19					**8**
*Acartia* spp.	9,10	11,14,15		9			**5**
*Microsetella norvegica*	6,9			9		30	**3**
*Paracalanus* spp.		14,15					**2**
*Calanus finmarchicus*						30	**1**
*Calocalanus styliremis*	25						**1**
*Centropages* spp.	20						**1**
Cyclopoida		12					**1**
*Detrichocoryceaus* spp.	10						**1**
*Metridia* spp.	9			9			**1**
*Microcalanus pygmaeus*				24			**1**
*Oithona atlantica*						30	**1**
*Oithona nana*	25						**1**
Oithonids		17					**1**
*Oncaea* spp.	29						**1**
Oncaeidae	10						**1**
*Pseudocalanus mimus*						30	**1**
*Temora* spp.	20						**1**
**Other zooplankton**	**AO**	**PO**	**IO**	**ArO**	**SO**	**Undet.**	**Total**
Appendicularians	3,8,9,10,21,25,27,29	2,11,14,15,17,18,19		9	7	30	**17**
*Oikopleura labradoriensis*						30	**1**
Doliolida	3,29	17,18					**4**
Pyrosomata		17,18,19					**3**
Salpida	3	18,19					**3**
Chaetognatha		14,16					**2**
Ctenophora		2					**1**
*Mertensia* spp. (Ctenophora)	20						**1**

Studies reporting misperformance (i.e. pronounced bias or a detection failure, as described in the Introduction section) of miniCOI metabarcoding with locally abundant taxa when compared against microscopy and/or another universal marker. Numbers indicate the code of the reference studies as in Tables S3 and S4. Misperformance reports are ranked by taxon and number of citations (copepods and other zooplankton categories) and grouped by ocean basin (AO, PO, IO, ArO and SO for Atlantic, Pacific, Indian, Arctic and Southern, respectively). Undeterminated location (Undet.) corresponds to study 30 (Zhang *et al.*, 2018) where authors constructed mock samples with individuals collected at `Canadian ports’ (with no further detail). Total: total number of studies reporting misperformance for each taxa. See Table S4 for full details of each article reviewed.

**Table III:** MiniCOI performance review synthesis-II.

Primers combination	Reviewed papers	Appendicularians				*Oithona similis*				Other taxa^*^
		Fail	Bias	N/A	OK	Fail	Bias	N/A	OK	
Leray–Folmer	3	2	0	1	0	2	0	1	0	Pronounced bias with *Acartia* spp.^11^
Leray–Meyer	2	1	0	1	0	0	1	1	0	Pronounced bias with *Oncaea* spp., failure to detect doliolids (Thaliacea)^29^. Pronounced bias or failure to detect *Centropages* spp. and *Temora* spp., failure to detect *Mertensia* spp. (Ctenophora)^20^
Leray–Geller	17	7	1	9	0	4	0	11	2	Pronounced bias with Cyclopoida^12^. Failure to detect Salpida (Thaliacea)^3,18–19^. Failure to detect Doliolida (Thaliacea)^3,17–18^. Failure to detect Pyrosomata (Thaliacea)^17–19^. Failure to detect *Microcalanus pygmaeus*^24^. Pronounced bias with chaetognaths^16^. Failure to detect oithonids^17^. Failure to detect Ctenophora^2^. Pronounced bias with *Acartia* spp. and *Paracalanus* spp.^14–15^. Failure to detect chaetognaths^14^
Leray XT	4	3	0	1	0	0	0	0	4	Pronounced bias with *Acartia* spp.^9–10^. Pronounced bias with *Detrichocoryceaus* spp. and Oncaeidae^10^. Pronounced bias with *Microsetella norvegica*^6,9^. Pronounced bias with *Metridia* spp.^9^
Other	4	3	0	1	0	0	1	3	0	Failure to detect *Calocalanus styliremis* and *Oithona nana*^25^*.* Failure to detect *Calanus finmarchicus*, *Pseudocalanus mimus*, *Microsetella norvegica*, *Oithona atlantica* and *Oikopleura labradonensis*^30^

Papers grouped by miniCOI primers combination (as in Table I). Summary on appendicularians and *Oithona similis*, and other locally abundant taxa, showing pronounced bias or a detection failure in the reviewed papers (*n* = 30). The total number of papers reviewed per miniCOI primers combination is shown. Fail: no detection (false negative); bias: pronounced bias (detected but at notably lower levels: more than an order of magnitude below in relative frequency that when compared to microscopy/alternative marker); N/A: either non field/mock presence or non-reported data; OK: rest of situations (= from fair to good correspondence with microscopy/alternative marker). See Table S4 for full details of each article reviewed.

^*^Other locally abundant taxa showing a detection failure or pronounced bias vs. microscopy and/or another universal marker. Superscripts indicate reference numbers as in Tables S3 and S4.

In total, misperformances were reported for 26 zooplankton taxa, including 18 copepods and five tunicates. Looking at the quotes mentioning either detection failures or pronounced biases for locally abundant taxa (Tables II and S4), two taxa stood out as the most cited ones: Class Appendicularia and the copepod species *Oithona similis*.

Apart from oithonids (and cyclopoids in general), a detection failure and/or a pronounced bias has been reported for copepod species from the genera *Acartia*, *Calanus*, *Calocalanus*, *Centropages*, *Detrichocorycaeus*, *Metridia*, *Microcalanus*, *Microsetella*, *Oncaea*, *Paracalanus*, *Pseudocalanus* and *Temora* (Tables II and III; see Table S4 for further details). Besides appendicularians, comparable biases have been reported for thaliaceans (salps, pyrosomes and doliolids), and for chaetognaths and ctenophores (Tables II and III; see Table S4 for further details). While extreme mitochondrial DNA (mtDNA) variability has been reported for both chaetognaths and ctenophores (Marlétaz *et al.*, 2017 and Pett *et al.*, 2011, respectively), which could explain the reported bias, hardly any mtDNA sequence is available for the abovementioned tunicate groups. Finally, the detection bias is usually reported at the genus or species level for copepods, whereas taxonomical levels over the Family are typical for the remaining taxa in this review, corresponding to a much-reduced number of specialized taxonomists.

The issues when detecting both appendicularians and *O. similis* were well supported by the bulk of the analyzed papers. It is to note that these are two zooplankton taxa with a well-known ecological importance and high abundance worldwide (e.g. Gallienne and Robins, 2001; Conley *et al.*, 2018). The reports of metabarcoding misperformance with appendicularians and *O. similis* encompassed the full time period reviewed, therefore suggesting a non-significant impact of reference databases’ depth for these species. We discuss both taxa independently as the nature and reach of the biases are different. Although we focus our primer mismatch analysis on *O. similis* and appendicularians, misperformances with other locally abundant taxa are briefly discussed below.

## COPEPODS

### Case study 1: *Oithona similis*

The cyclopoid copepod *O. similis* has been proposed as the most abundant copepod on our planet and inhabits tropical to boreal regions (Gallienne and Robins, 2001). However, as reported for other copepod species with a cosmopolitan distribution, it is known to harbor different lineages that could correspond to cryptic species or pseudo-species (Cornils *et al.*, 2017). For this taxon, our analyses showed a contrasting performance of DNA metabarcoding, ranging from good correspondence to detection failure, when compared with either microscopy and/or other universal markers (Table III; see Table S4 for further details). In detail, *O. similis* was reported in fourteen of the reviewed papers, and for eight of them, authors reported either the failure of detection or a pronouncedly biased one (six and two articles, respectively). Interestingly, a fair/good correspondence was only reported for the two most degenerated primers, with a 100% of success in the Leray XT ones (four works), and a 29% in the Leray–Geller ones (two out of seven studies, including Schroeder *et al.*, 2020, that used a cocktail of primers).

**Fig. 4:**
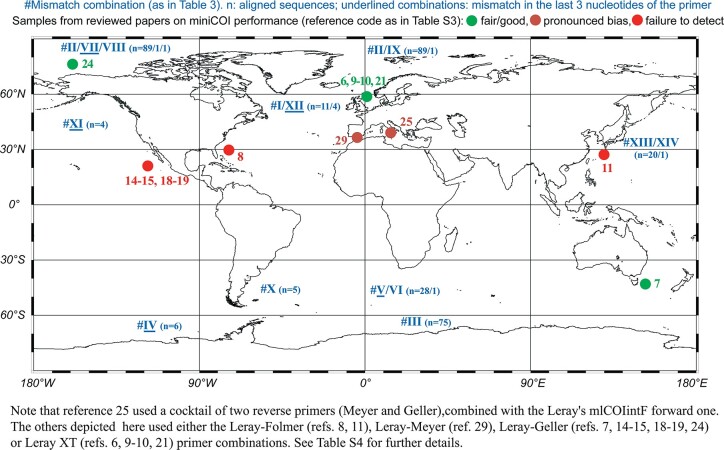
Map of studies where DNA metabarcoding performance with *Oithona similis* is reviewed (see Tables II and S4 for performance details).

The different miniCOI primer combinations showed a distinct performance, and this also applied when using the same primer combination in different geographical areas (Fig. 4, Table S4). Because of this, we investigated the number of primer mismatches along the geographical range of this taxon. With this aim, we downloaded all the available *O. similis* COI region sequences in the MZG database (as of 9 January 2024). We filtered sequences that failed to align and those not including the miniCOI region, ending up with an alignment of 247 sequences (accession numbers in Table IV). Due to its terminal location in the Folmer *et al.* (1994) fragment, only five sequences out of 247 covered the whole reverse primer region. However, the 247 sequences contained the whole forward primer region allowing us to study the mismatches for this region.

### Analysis of forward primer region

We observed eight variable (non-conserved) positions for *O. similis* in the 26 bp long miniCOI forward primer region (corresponding to every third codon positions). We analyzed the frequency and location of the mismatches, taking into account the nature of the ambiguities within the two most common miniCOI forward primers (Leray’s mlCOIintF and Leray XT; Table S5). Six variable positions showed the same mismatch frequency for both primers, due to shared ambiguities (with mismatches affecting from none to up to 86 of the 247 aligned sequences), including one in the third nucleotide from the 3′ end for 17% of the sequences. However, a distinct impact corresponded to the remaining two variable positions (those at the 12^th^ and 18^th^ nucleotide). While the Leray XT primer would perfectly match both positions for the 247 aligned sequences, this would only apply to a fraction of them for the Leray’s mlCOIintF primer (123 and 23 sequences, respectively; Table S5).

### The cryptic nature of *O. similis* explains the contrasting performance of miniCOI metabarcoding worldwide

Arranging the 247 *O. similis* sequences by the different combinations of nucleotides at the eight variable positions in the forward primer region, we encountered fourteen different combinations (lineages #I to #XIV as in Table IV) that corresponded to different geographical origins of the samples. Therefore, we checked whether this geographical genetic variability, which results in a different number of mismatches in the forward primer region, could explain the reported contrasting performance of miniCOI metabarcoding for *O. similis* worldwide (Fig. 4). On one hand, the total number of mismatches for the forward primer region ranged from one to six for the Leray’s mlCOIintF, but from none to four for the Leray XT (Table IV). On the other hand, five out of the 14 lineages included a mismatch in the third nucleotide from the 3′ end, affecting both primers equally (Table IV).

The best *O. similis* detection performances were reported for the most degenerated Leray XT primers (Fig. 4), corresponding to a low number of mismatches with the miniCOI forward primer (Table IV). In addition, a detection failure (red dots in Fig. 4) was consistently reported for this species when applying the most commonly used primers (Leray–Geller) in the North–Western Pacific area (Harvey *et al.*, 2017, 2018; Matthews and Blanco-Bercial, 2023; Matthews and Ohman, 2023), where the taxon showed the highest number of mismatches for the forward primer (Table IV). Other works applying the Leray–Geller primers reported a fair–good correspondence (Deagle *et al.*, 2018; Questel *et al.*, 2021) or a pronounced bias (but capable of detecting the taxon; Schroeder *et al.*, 2020) in geographical areas including *O. similis* lineages with less primer mismatches (green and orange asterisks; Fig. 4, Table IV). Although a geographical correspondence of contrasting performances with DNA metabarcoding and the distinct mismatches number of *O. similis*’ lineages is evident, we cannot be certain of the *O. similis* lineage/s sampled in each of the reviewed studies. It is important to note that for some areas more than one lineage (with distinct mismatch combinations) could co-exist.

**Table IV:** Mismatches in O. similis for miniCOI forward primer.

Combination	*n*	Area	Cornils *et al.* (2017) lineage	Acc. numbers	5′ location of eight non-conserved positions (see Table S5)								Mismatches mlCOIintF/Leray XT
					#3	#6	#9	#12	#15	#18	#21	#24	
#I	11	North Sea, Mediterranean	NA (North Atlantic)	KU982714–16, KU982891–98	**C**	A	T	A	T	T	C	C	1/1
#II	89	Arctic Ocean, Norwegian Sea	ARK (Arctic)	KU982717–79,KU982859–61, KU982864–65, KU982867–81, KU982899, KU982918–20, KU982922, KU982925	T	A	T	*G*	T	*G*	C	C	2/0
#III	75	Southern Ocean	ANT (Antarctic)	KU982780–2830, KU982834, KU982884–90, KU982900–13, KU982915–16	T	**C**	T	A	T	*G*	C	T	2/1
#IV	6	Southern Ocean	AS (Amundsen Sea)	KU982831–33, KU982934–36	T	**G**	T	A	T	T	C	**A**	2/2
#V	28	Southern Ocean	PF (Polar Front)	KU982835–58, KU982882, KU982917, KC754452–53	**C**	A	T	A	T	*G*	T	**A**	3/2
#VI	1	Southern Ocean	PF (polar Front)	KU982914	T	T	T	A	T	*G*	C	T	1/0
#VII	1	Arctic Ocean	ARK2 (Arctic: Chukchi Plateau)	KU982926	**G**	T	**C**	A	**G**	A	C	**A**	4/4
#VIII	1	Arctic Ocean	ARK3 (Arctic: Chukchi Plateau)	KU982927	T	A	T	A	T	*G*	T	C	1/0
#IX	1	Arctic Ocean	ARK (Arctic: Greenland Sea)	KU982928	T	**G**	T	*G*	T	*G*	C	C	3/1
#X	5	Beagle Channel (Argentina)	BC (Beagle Channel)	KU982929–33	**C**	A	T	*G*	T	*G*	C	C	3/1
#XI	4	Canada (Pacific Ocean), California	New sequences, from MZGdb^*^	CAISN1378–13, CAISN218–12, CAISN231–12, ZPC049–13	**G**	**G**	**C**	*G*	A	*G*	C	**A**	6/4
#XII	4	Canada (Hudson Bay), North Sea	New sequences, from MZGdb^*^	CAISN326–12, KT208459, KT208745, MG316221	**C**	T	T	*G*	T	T	C	**G**	3/2
#XIII	20	Pacific Ocean (China, Korea and 1 N/A)	NP (North Pacific), plus KR048987 from MZGdb^*^	EU599542–44, JF269185–86, JN230859–71, KC287768, KR048987	T	A	T	*G*	A	*G*	T	C	2/0
#XIV	1	N/A (probably Korea)	New sequences, from MZGdb^*^	KR048986	**C**	A	T	*G*	A	A	T	C	2/1

Different combinations of nucleotides in *O. similis* for the eight non-conserved positions of the miniCOI forward primer region (see Table S5 for the whole forward primer). Note that site #24 corresponds to the third nucleotide from the 3′ end. *n*: number of sequences; nucleotides in italics: mismatch with Leray’s mlCOIintF forward primer; nucleotides in bold: mismatch with both Leray’s mlCOIintF and Leray XT forward primers. Number of total mismatches with each of the primers is given in the last column. GenBank accession numbers are also shown. ^*^New sequences not from Cornils *et al.* (2017), downloaded for this work from MZGdb.

The most degenerated Leray XT primers would be superior regarding PCR amplification success. However, these primers have only been tested so far in zooplankton samples from Northern European waters (Fig. 4, Table S4). As *O. similis* lineages reported for these areas present a relatively low number of mismatches in the forward primer region (Table IV), both factors might be contributing to the fair to good correspondence reported for this taxon in the works applying the Leray XT primers (Coguiec *et al.*, 2021; Ershova *et al.*, 2021, 2023; Ohnesorge *et al.*, 2023). Although the use of Leray XT primers could prevent or improve metabarcoding misperformance for certain *O. similis* lineages, this would not be the case for all of them. For example, a maximum of four mismatches for the Leray XT forward primer was present in lineages #VII and #XI, including one mismatch in the third nucleotide from the 3′ end in both (Table IV). The higher degeneracy of the Leray XT primers also has the trade-off of more non-target species amplification, such as bacteria, thus reducing coverage (e.g. Collins *et al.*, 2019; Hintikka *et al.*, 2022). This might be a serious limitation when metabarcoding samples with relatively low eukaryote proportion such as environmental DNA *sensu stricto* (eDNAss), but might not be so relevant when using zooplankton from net samples.

### Case study 2: *Oithona nana*

MiniCOI detection issues with cyclopoids have been reported elsewhere (see Table II; including other locally abundant *Oithona* species such as *O. nana* or *O. atlantica*, Table S4). This could correspond to a Cyclopoida general failure with the COI region (e.g. Cepeda *et al.*, 2012; Jungbluth *et al.*, 2021). For this reason, we performed the previous mismatch analysis to the congeneric *O. nana*. Results with the miniCOI forward primer region identified two haplotypes (“A” and “B” in Table S6), although with a much lower *n* when compared to *O. similis* (16 *O. nana* sequences). While for the “A” haplotype there were two and one mismatches, for the Leray’s mlCOIintF and Leray XT primers, respectively, both primers would present five mismatches on haplotype “B”, including one in the third nucleotide from the 3’ end, pointing to an amplification failure.

### Case study 3: *Microsetella norvegica*

Misperformances have been reported even for the most degenerate Leray XT primers with other copepod species. These include *Oncaea*, *Corycaeus*, *Microsetella* and some *Acartia* species (Table III). As an example, we analyzed mismatches for genus *Microsetella.* While Coguiec *et al.* (2021) and Ershova *et al.* (2021) reported a misperformance with Leray XT primers, Zhang *et al.* (2018) reported a detection failure with two different miniCOI barcodes, and we also found a detection failure using the Leray–Meyer primer combination (Bay of Biscay samples; Aitor Albaina, pers. comm.). There were two *Microsetella* species in the MZG database, but just two COI sequences for *M. rosea* so we focused on *M. norvegica* (*n* = 24). With this species, the number and location of the mismatches in the forward primer region also point to an amplification failure. More specifically, five and four mismatches were found in most of the sequences (19 out of 24) for the Leray’s mlCOIintF and Leray XT primers, respectively, and in both cases including a mismatch in the third nucleotide from the 3′ end (Table S7).

## TUNICATES

### Case study 4: the intriguing case of Class Appendicularia

Appendicularians are an important group of zooplankton, playing a critical role in the marine food web due to both their abundance and trophic niche (e.g. Gorsky and Fenaux, 1998; Conley *et al.*, 2018), which so far has consistently failed to be amplified by COI metabarcoding. In the present review, all the miniCOI primers combinations failed to detect appendicularians, except for a sole paper (see Tables III and S4 for further details). In that study, Matthews *et al.* (2021) reported a pronounced bias with this taxon when comparing miniCOI to both 18S V4 metabarcoding and image based analysis. However, Matthews and Ohman (2023) failed to detect them in the same ecosystem when they applied 18S V4 metabarcoding in a media with abundant appendicularians. The reasons why appendicularians detection consistently fails are two-fold.

On the one side, only few sequences of appendicularian miniCOI region have been published and only of four species: *Oikopleura dioica*, from expressed sequence tag sequencing (Denoeud *et al.*, 2010; Wang *et al.*, 2015), *O. longicauda* (Sakaguchi *et al.*, 2017; Naville *et al.*, 2019), *Bathochordaeus stygius* and *Mesochordaeus erythrocephalus* (Naville *et al.*, 2019), from shotgun sequencing (Table S9). *O. dioica* and *O. longicauda* are common coastal species (Fenaux, 1963), while *Bathochordaeus* spp. and *Mesochordaeus erythrocephalus* are deep sea species not likely to be frequently encountered during metabarcoding studies (Hopcroft and Robison, 1999; Sherlock *et al.*, 2017). However, although the low availability of reference sequences would prevent a high taxonomic resolution with appendicularians, it would not prevent PCR amplification. In this regard, Matthews and Blanco-Bercial (2023) reported miniCOI (Leray–Geller) primers failing to amplify (in individual PCR reactions) the seven appendicularian species they tried from the California current system.

### The role of poly-T inserts in appendicularians

**Fig. 5:**
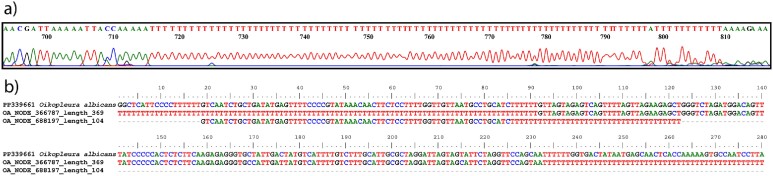
Poly-T inserts in Appendicularia. (**a**) Chromatogram showing poly-T section in the coding strand of mitochondrial DNA of an appendicularian (*Appendicularia sicula*); (**b**) alignment of *Oikopleura albicans* COI sequence obtained by Sanger sequencing from complementary DNA (i.e. after reverse transcription from messenger RNA) and typical COI fragments of genomic mtDNA flanked by poly-T regions obtained by Illumina sequencing (courtesy of Carmela Gissi, University of Bari, Italy). See Table S9 for further details.

On the other side, our analysis indicate that the miniCOI amplification failure in appendicularians would result from a combination of mismatches in the forward primer region and the presence of homopolymeric poly-T inserts in the COI region of several species (Table V). These were first discovered in *O. dioica* (Denoeud *et al.*, 2010). They are unusual intron-like inserts, which consist of tens or hundreds of consecutive Ts in the coding strand (poly-As in non-coding strand, Fig. 5; Dierckxsens *et al.*, 2024; Klirs *et al.*, 2024). These homopolymeric introns are excised after DNA is copied into messenger RNA (mRNA). The potential reason for their existence is unknown, as is the mechanism of excision and enzyme machinery involved in the process. It has been postulated that poly-T inserts are likely corrected by RNA editing, rather than by an intron-like splicing, since potential intermediates of excision process were detected in ESTs (Denoeud *et al.*, 2010). According to Latour (2021), *Oikopleura vanhoeffeni* also contains poly-T inserts in its mtDNA. The size of poly-T inserts in *O. vanhoeffeni* can be up to ~ 1 500 bp long and is highly variable between individuals. Unfortunately, *O. vanhoeffeni* COI sequences are not yet publicly available. In *O. dioica* the homopolymers are up to 500 bp long with an average length of about 140 bp (Klirs *et al.*, 2024). Poly-T inserts, because of their frequency and length, make it practically impossible to sequence mtDNA using the Sanger method or Illumina sequencing (Denoeud *et al.*, 2010; Dierckxsens *et al.*, 2024; Klirs *et al.*, 2024). Based on the available *O. longicauda*, *Bathochordaeus* and *Mesochordaeus* sequences in GenBank, it is obvious that not all appendicularians possess poly-T inserts in their mtDNA.

**Table V:** Mismatches in Class Appendicularia for the miniCOI forward primer.

		5′ location of 14 non-conserved positions														Mismatches mlCOIintF/Leray XT	Poly-Ts
Acc. numbers	Species	#1	#3	#4	#5	#6	#7	#9	#12	#15	#16	#18	#22	#23	#24		
SCLE01415711	*Bathochordaeus stygius*	**A**		**T**			*A*		*G*		*A*				**A**	6/3	
SCLF01725989	*Mesochordaeus erythrocephalus*	**A**		**T**			*A*	**G**	*G*		*A*					6/3	
PP339655	*Megalocercus abyssorum*	**A**		**T**		**C**	*A*	**G**	*G*		*A*					7/4	
LC222754	*Oikopleura longicauda*			**T**			*A*	**G**	*G*		*A*					5/2	
SCLD01101138	*O. longicauda*			**T**			*A*	**G**	*G*		*A*					5/2	
PP339656	*O. longicauda*			**T**					*G*		*A*					3/1	
PP339657	*O. longicauda*		**C**	**T**					*G*	**C**	*A*				**A**	6/4	
PP339658	*O. longicauda*	**A**		**T**					*G*		*A*					4/2	
PP339659	*O. fusiformis*		**C**	**T**					*G*	**G**	*A*	*G*	**G**	**T**		8/5	
SAMN00177767	*O. dioica*	**A**		**T**			*A*		*G*	**C**	*A*					6/3	X
PP339660	*O. dioica*	**A**		**T**			*A*			**C**	*A*					5/3	X
GCJN01047493	*O. dioica*	**A**		**T**					*G*		*A*					4/2	X
PP339661	*O. albicans*		**G**	**T**			*A*		*G*		*G*					5/2	X
PP339662	*Stegosoma magnum*	**A**		**T**			*A*		*G*		*A*	*C*				6/2	X
PP339663	*Appendicularia sicula*	**A**														1/1	X
PP339664	*Fritillaria borealis sargassi*	**A**									*T*	*G*				3/1	X
PP339665	*F. pellucida*	**A**	**G**				*A*				*T*				**A**	5/3	X
PP339666	*F. formica tuberculata*	**A**		**T**	**G**											3/3	X
PP339667	*Kowalevskia tenuis*	**A**	**G**	**G**	**G**	**G**		**G**			*T*					7/6	
PP339668	*K. oceanica*	**A**		**G**	**G**	**G**										4/4	
PP339669	*K. oceanica*	**A**		**G**	**G**	**G**										4/4	

MiniCOI forward primer mismatch analysis in Appendicularia for the fourteen non-conserved positions. Note that sites #22 to #24 correspond to the fifth to third nucleotides from the 3′ end. Nucleotides in italics: mismatch with Leray’s mlCOIintF forward primer; nucleotides in bold: mismatch with both Leray’s mlCOIintF and Leray XT forward primers. Number of total mismatches with each of the primers is given. The last column shows which appendicularian species contain poly-T inserts in their mitochondrial DNA (crosses) and those that do not (empty).

To obtain more information on the presence of poly-T inserts in Class Appendicularia, we amplified the COI region of different appendicularian species (Tables S8 and S9), either from mtDNA or, when not possible (due to the presence of the inserts), from complementary DNA (i.e. after reverse transcription from mRNA, see Table S9 for details and Fig. 5). We obtained 15 new COI sequences from 12 appendicularian species (including 10 species in which COI was sequenced for the first time; Table S9). This dataset was supplemented by six sequences obtained from GenBank (adding two species to the previous ones).

### Primer mismatch analysis

The primer mismatch analysis showed that the problematic primer is the forward one of the miniCOI region (Table V), as shown earlier with *O. similis*, while the reverse primer is almost without mismatches (Table S10). This agrees with the more conserved nature of the reverse primer region (Folmer’s HCO 2198) shown across animals by Sharma and Kobayashi (2014). It is of note that the mismatches in the forward primer region in appendicularians were not restricted to the third positions of codons, as in *O. similis.* Thus, complicating even more the design of primer binding sites within this Class (Table V). Only four appendicularian species, out of the 14 analyzed, contained fewer than four mismatches with the Leray’s mlCOIintF primer (this increasing to ten species when considering the Leray XT one). Seven species contained poly-T inserts within the miniCOI. This includes all the species showing less than four mismatches for the mlCOIintF primer, except for *O. longicauda* (but see below), pointing to a general metabarcoding amplification failure with this Class (Table V). Therefore, the only common species likely to be recovered in miniCOI metabarcoding studies, at least for some regional haplotypes, would be *O. longicauda* (Table V). However, another problem arises with *O. longicauda*, which is the high intraspecific variability of COI sequences, suggesting multiple cryptic species/pseudo-species. The three Mediterranean *O. longicauda* individuals (accession numbers PP339656-8) differed in between 22% and 26% of the nucleotides for the whole COI region among themselves, and between 19% and 26% when compared to the Pacific Ocean sequences (SCLD01101138 and LC222754).

### Case study 5: Class Thaliacea

We have found that the detection failure seems to be affecting the sister Class Thaliacea (salps, doliolids and pyrosomes) as well (Table II). We applied a primer mismatch analysis to 12 species whose COI sequences were available in GenBank (six doliolids, five salps and one pyrosome; Table S11). Similarly to Appendicularia, the reverse primer jgHCO2198 is almost a perfect match (Table S12). However, this did not apply to the forward primer region, where up to eight and seven mismatches are reported for the Leray’s mlCOIintF and Leray XT primers, respectively (Table VI), with doliolids having the least number of mismatches. The reduced number of reference sequences (Yokobori *et al.*, 2005; Garić and Batistić, 2016; Goodall-Copestake, 2018; Garić and Batistić, 2022) and the high number of mismatches with the forward primers would explain the detection issues with Thaliaceans (Tables II and S4).

**Table VI:** Mismatches in Class Thaliacea for the miniCOI forward primer.

		5′ location of 13 non-conserved positions													Mismatches mlCOIintF/Leray XT
Acc. numbers	Species	#1	#2	#3	#4	#5	#6	#7	#9	#12	#15	#16	#18	#24	
OP437494	*Dolioletta advena*	**A**						*A*							2/1
OP437493	*D. advena*	**A**						*A*			**G**				3/2
OP437492	*D. advena*	**A**						*A*							2/1
OP437491	*D. gegenbauri*	**A**						*A*	**G**	*G*					4/2
OP437490	*Doliolina krohni*	**A**					**G**			*G*					3/2
OP437489	*D. muelleri*	**A**						*A*			**G**		*C*		4/2
OP437495	*Doliolum nationalis*			**G**				*A*	**G**	*G*			*C*		5/2
AB176541	*D. nationalis*			**G**				*A*	**G**	*G*			*C*		5/2
OP437487	*D. denticulatum*	**A**						*A*	**G**	*G*	**G**				5/3
OP437488	*Pyrosoma atlanticum*	**A**						*A*	**G**		**C**		*G*	**A**	6/4
MT998285	*Thalia longicauda*	**T**	**C**		**T**	**G**		*A*			**C**	*A*	*C*		8/5
MH626415	*T. democratica*	**T**	**C**		**T**	**G**		*A*	**G**			*A*			7/5
KT818686	*Brooksia lacromae*	**T**	**C**		**T**	**G**		*A*				*A*	*C*		7/4
LC333181	*Salpa fusiformis*	**T**	**C**		**T**	**G**				*G*		*A*			6/4
LC333180	*S. thompsoni*	**T**	**C**		**T**	**G**		*A*				*A*		**A**	7/5

MiniCOI forward primer mismatch analysis in Thaliacea for the 13 non-conserved positions. Note that site #24 corresponds to the third nucleotide from the 3′ end. Nucleotides in italics: mismatch with Leray’s mlCOIintF forward primer; nucleotides in bold: mismatch with both Leray’s mlCOIintF and Leray XT forward primers. Number of total mismatches with each of the primers is given.

## CONCLUDING REMARKS

Misperformances with miniCOI metabarcoding, either detection failures (i.e. false negatives) or pronounced biases, have been reported for at least 26 zooplankton taxa, including 18 copepods and five tunicates (Table II). According to our data, the most commonly cited taxa suffering from these biases were the copepod *Oithona similis* and the Class Appendicularia, two globally abundant taxa. Our results with key zooplankton species confirm the previous suggestion about the constraints of COI for the assessment of the whole eukaryotic community (e.g. Clarke *et al.*, 2014; Deagle *et al.*, 2014; Piñol *et al.*, 2015). However, due to the demonstrated high taxonomic resolution and the increasing depth of the reference databases (Andújar *et al.*, 2018), we support the continued use of COI for zooplankton biodiversity monitoring. In this regard, we strongly recommend the use of miniCOI in combination with at least one more conserved universal primer and/or morphological analysis methods, able to detect oithonids and appendicularians, among other groups overlooked by miniCOI.

Although primer mismatches in the forward primer region arose as a key factor explaining misperformance with the analyzed taxa, other factors cannot be discarded. While we did not account for different DNA extraction methods, PCR/library construction settings or bioinformatics pipelines (already reviewed by Santoferrara, 2019 and Laakmann *et al.*, 2020 for plankton samples), the fact that results are consistent along the reviewed period (2017 to 2023) supports the reported pattern for *O. similis* and appendicularians.

Contrasting performances with *O. similis* worldwide would be associated with non-optimum amplification due to a different number of mismatches in the miniCOI forward primers, corresponding to the intraspecific variability for this taxon (Cornils *et al.*, 2017). This limitation is somewhat overcome, for some regions/lineages, by applying the more degenerated Leray XT combination. However, using the Leray XT primers comes with a trade-off of more non-target taxa amplification thus reducing sequencing coverage (Collins *et al.*, 2019; Hintikka *et al.*, 2022). Besides, more degenerate primers also pose the limitation of not allowing the use of high fidelity polymerases (e.g. Jungbluth *et al.*, 2021).

Failure to detect appendicularians is explained by the combination of a generalized high number of mismatches in the forward primer region and the presence of poly-T inserts in several species. Moreover, the scarcity of taxonomists capable of identifying species under the microscope along with the lack of reference sequences further complicates the correct assessment of this Class. Due to this, we recommend targeting other genome fragments for this group. Besides the 18S rRNA regions capable of detecting appendicularians (at least at Class level: V1–2, V4 and V9; Table S4), the internal transcribed spacer (ITS) located also in the rRNA, represents a promising alternative for this group (ITS1-5.8S and ITS2-28S fragments; Garić *et al.*, 2018 and Masunaga *et al.*, 2022). When considering the detection of other gelatinous groups (such as Thaliacea or Ctenophora), where biases with miniCOI have also been reported (Table II), 18S rRNA fragments are also deemed superior compared to COI ones (e.g. Ruiz *et al.*, 2024).

Despite the fact that several studies have already compared miniCOI metabarcoding vs. microscopy and/or other universal markers with zooplankton samples, more studies are needed to anticipate and unveil false negatives as well as pronounced amplification biases with local key species when applying this marker. Furthermore, an in-depth analysis of the limitations of other universal markers worldwide would be convenient to tackle the high genetic diversity within the marine zooplankton communities. In fact, given the distinct nature of PCR amplification biases for different zooplankton groups and the critical nature of local variability (lineage-dependent) within certain species, we call for the combination of (i) several amplicons (aiming for complementarity and, ideally, including group-specific primers along with more universal ones as to cover the bulk of the community) and (ii) local reference databases (including, at least, those from locally abundant species/lineages). Without this, the putative semiquantitative value of DNA metabarcoding (e.g. Lamb *et al.*, 2019) would be highly compromised. In this regard, recent works support the calibration of metabarcoding-based quantitative data (relative abundances) by identifying and correcting PCR amplification biases in complex community samples (McLaren *et al.*, 2019; Shelton *et al.*, 2023). The use of mock DNA samples with a known composition, including the locally key species/lineages along with enough replicates, seems promising when correcting bias in quantitative metabarcoding (Shelton *et al.*, 2023). Besides, standardization of primers and databases used would facilitate comparisons among researches and ecosystems. Finally, since all zooplankton identification methods, including the variety of DNA fragments proposed and morphological taxonomy, present both pros and cons, an integrative taxonomic approach is needed.

## Supplementary Material

Figure-S1-Albaina-etal300_fbae057

Figure_S2_Albaina_etal_revised300_fbae057

SupplementaryTables_Albainaetal_revised_fbae057

TableS4_Albaina_etal_R2_fbae057

## Data Availability

New DNA sequences associated with this article are available in GenBank, accession numbers PP339655-PP339669.
