# Supplementary figures and images for: Know your limits; miniCOI metabarcoding fails with key marine zooplankton taxa
Source: J Plankton Res. 2024 Nov 2;46(6):581–95. doi: 10.1093/plankt/fbae057 (PMC11629781; doi:10.1093/plankt/fbae057)

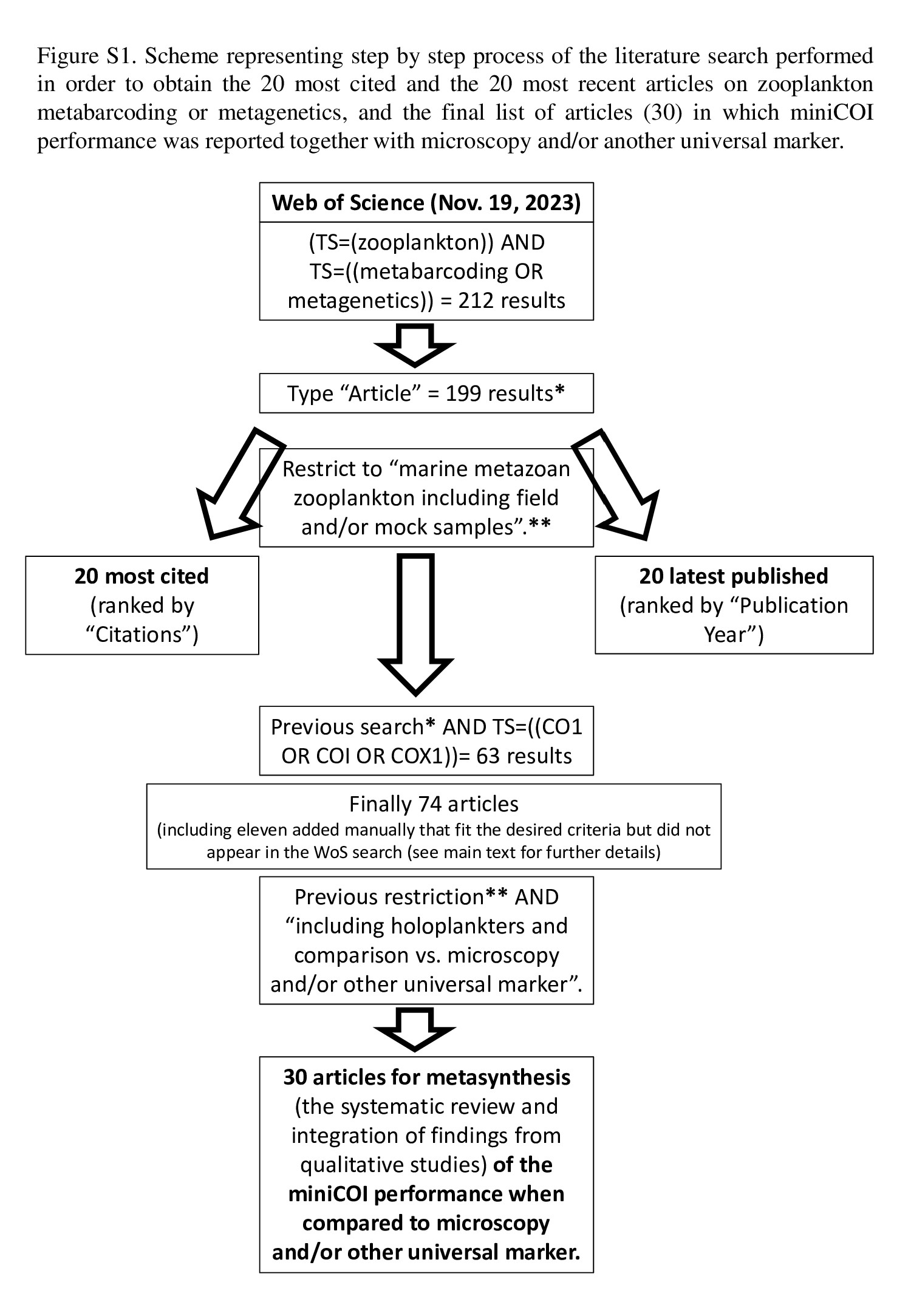

Supplement: Figure-S1-Albaina-etal300_fbae057 [file figure-s1-albaina-etal300_fbae057.jpeg]

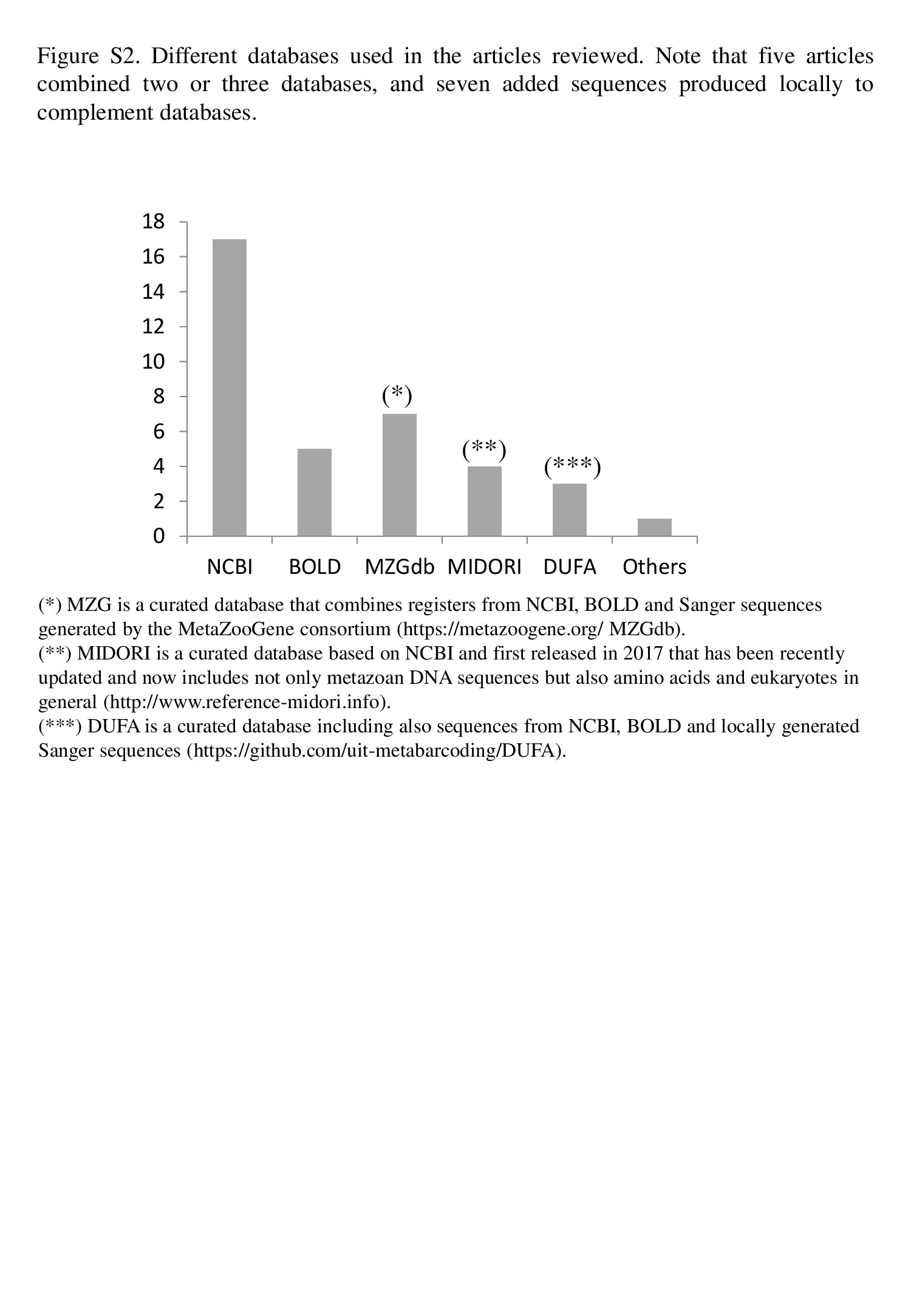

Supplement: Figure_S2_Albaina_etal_revised300_fbae057 [file figure_s2_albaina_etal_revised300_fbae057.jpeg]
