# Supplementary material for: Know your limits; miniCOI metabarcoding fails with key marine zooplankton taxa
Source: J Plankton Res. 2024 Nov 2;46(6):581–95. doi: 10.1093/plankt/fbae057 (PMC11629781; doi:10.1093/plankt/fbae057)
Supplement: SupplementaryTables_Albainaetal_revised_fbae057 [file supplementarytables_albainaetal_revised_fbae057.docx]

***Supplementary tables. Albaina et al.***

Table S1. Relation of the 20 most cited articles on zooplankton metabarcoding or metagenetics. See text and Fig. S1 for further details.

1. Abad, D., Albaina, A., Aguirre, M., Laza-Martínez, A., Uriarte, I., Iriarte, A., Villate, F., and Estonba, A. (2016) Is metabarcoding suitable for estuarine plankton monitoring? A comparative study with microscopy. *Marine Biology*, **163**, 149. [10.1007/s00227-016-2920-0](https://doi.org/10.1007/s00227-016-2920-0).
2. Brown, E. A., Chain, F. J. J., Crease, T. J., MacIsaac, H. J., and Cristescu, M. E. (2015) Divergence thresholds and divergent biodiversity estimates: can metabarcoding reliably describe zooplankton communities? *Ecology and Evolution*, **5**, 2234–2251. [10.1002/ece3.1485](https://doi.org/10.1002/ece3.1485).
3. Brown, E. A., Chain, F. J. J., Zhan, A., MacIsaac, H. J., and Cristescu, M. E. (2016) Early detection of aquatic invaders using metabarcoding reveals a high number of non-indigenous species in Canadian ports. *Diversity and Distributions*, **22**, 1045–1059. [10.1111/ddi.12465](https://doi.org/10.1111/ddi.12465).
4. Bucklin, A., Yeh, H. D., Questel, J. M., Richardson, D. E., Reese, B., Copley, N. J., and Wiebe, P. H. (2019) Time-series metabarcoding analysis of zooplankton diversity of the NW Atlantic continental shelf. *ICES Journal of Marine Science*, **76**, 1162–1176. [10.1093/icesjms/fsz021](https://doi.org/10.1093/icesjms/fsz021).
5. Carroll, E. L., Gallego, R., Sewell, M. A., Zeldis, J., Ranjard, L., Ross, H. A., Tooman, L. K., O’Rorke, R., *et al.* (2019) Multi-locus DNA metabarcoding of zooplankton communities and scat reveal trophic interactions of a generalist predator. *Scientific Reports*, **9**, 281. [10.1038/s41598-018-36478-x](https://doi.org/10.1038/s41598-018-36478-x).
6. Chain, F. J. J., Brown, E. A., MacIsaac, H. J., and Cristescu, M. E. (2016) Metabarcoding reveals strong spatial structure and temporal turnover of zooplankton communities among marine and freshwater ports. *Diversity and Distributions*, **22**, 493–504. [10.1111/ddi.12427](https://doi.org/10.1111/ddi.12427).
7. Clarke, L. J., Beard, J. M., Swadling, K. M., and Deagle, B. E. (2017) Effect of marker choice and thermal cycling protocol on zooplankton DNA metabarcoding studies. *Ecology and Evolution*, **7**, 873–883. [10.1002/ece3.2667](https://doi.org/10.1002/ece3.2667).
8. Darling, J. A., Martinson, J., Gong, Y., Okum, S., Pilgrim, E., Lohan, K. M. P., Carney, K. J., and Ruiz, G. M. (2018) Ballast Water Exchange and Invasion Risk Posed by Intracoastal Vessel Traffic: An Evaluation Using High Throughput Sequencing. *Environ. Sci. Technol.*, **52**, 9926–9936. [10.1021/acs.est.8b02108](https://doi.org/10.1021/acs.est.8b02108).
9. Deagle, B. E., Clarke, L. J., Kitchener, J. A., Polanowski, A. M., and Davidson, A. T. (2018) Genetic monitoring of open ocean biodiversity: An evaluation of DNA metabarcoding for processing continuous plankton recorder samples. *Molecular Ecology Resources*, **18**, 391–406. [10.1111/1755-0998.12740](https://doi.org/10.1111/1755-0998.12740).
10. Djurhuus, A., Pitz, K., Sawaya, N. A., Rojas-Márquez, J., Michaud, B., Montes, E., Muller-Karger, F., and Breitbart, M. (2018) Evaluation of marine zooplankton community structure through environmental DNA metabarcoding. *Limnology and Oceanography: Methods*, **16**, 209–221. [10.1002/lom3.10237](https://doi.org/10.1002/lom3.10237).
11. Ershova, E. A., Descoteaux, R., Wangensteen, O. S., Iken, K., Hopcroft, R. R., Smoot, C., Grebmeier, J. M., and Bluhm, B. A. (2019) Diversity and Distribution of Meroplanktonic Larvae in the Pacific Arctic and Connectivity With Adult Benthic Invertebrate Communities. *Frontiers in Marine Science*, **6**.
12. Flynn, J. M., Brown, E. A., Chain, F. J. J., MacIsaac, H. J., and Cristescu, M. E. (2015) Toward accurate molecular identification of species in complex environmental samples: testing the performance of sequence filtering and clustering methods. *Ecology and Evolution*, **5**, 2252–2266. [10.1002/ece3.1497](https://doi.org/10.1002/ece3.1497).
13. Lacoursière-Roussel, A., Howland, K., Normandeau, E., Grey, E. K., Archambault, P., Deiner, K., Lodge, D. M., Hernandez, C., *et al.* (2018) eDNA metabarcoding as a new surveillance approach for coastal Arctic biodiversity. *Ecology and Evolution*, **8**, 7763–7777. [10.1002/ece3.4213](https://doi.org/10.1002/ece3.4213).
14. Lindeque, P. K., Parry, H. E., Harmer, R. A., Somerfield, P. J., and Atkinson, A. (2013) Next Generation Sequencing Reveals the Hidden Diversity of Zooplankton Assemblages. *PLOS ONE*, **8**, e81327. [10.1371/journal.pone.0081327](https://doi.org/10.1371/journal.pone.0081327).
15. Mohrbeck, I., Raupach, M. J., Martínez Arbizu, P., Knebelsberger, T., and Laakmann, S. (2015) High-Throughput Sequencing—The Key to Rapid Biodiversity Assessment of Marine Metazoa? *PLOS ONE*, **10**, e0140342. [10.1371/journal.pone.0140342](https://doi.org/10.1371/journal.pone.0140342).
16. Schroeder, A., Stanković, D., Pallavicini, A., Gionechetti, F., Pansera, M., and Camatti, E. (2020) DNA metabarcoding and morphological analysis - Assessment of zooplankton biodiversity in transitional waters. *Marine Environmental Research*, **160**, 104946. [10.1016/j.marenvres.2020.104946](https://doi.org/10.1016/j.marenvres.2020.104946).
17. Sommer, S. A., Van Woudenberg, L., Lenz, P. H., Cepeda, G., and Goetze, E. (2017) Vertical gradients in species richness and community composition across the twilight zone in the North Pacific Subtropical Gyre. *Molecular Ecology*, **26**, 6136–6156. [10.1111/mec.14286](https://doi.org/10.1111/mec.14286).
18. Stefanni, S., Stanković, D., Borme, D., de Olazabal, A., Juretić, T., Pallavicini, A., and Tirelli, V. (2018) Multi-marker metabarcoding approach to study mesozooplankton at basin scale. *Scientific Reports*, **8**, 12085. [10.1038/s41598-018-30157-7](https://doi.org/10.1038/s41598-018-30157-7).
19. Zaiko, A., Samuiloviene, A., Ardura, A., and Garcia-Vazquez, E. (2015) Metabarcoding approach for nonindigenous species surveillance in marine coastal waters. *Marine Pollution Bulletin*, **100**, 53–59. [10.1016/j.marpolbul.2015.09.030](https://doi.org/10.1016/j.marpolbul.2015.09.030).
20. Zhang, G. K., Chain, F. J. J., Abbott, C. L., and Cristescu, M. E. (2018) Metabarcoding using multiplexed markers increases species detection in complex zooplankton communities. *Evolutionary Applications*, **11**, 1901–1914. [10.1111/eva.12694](https://doi.org/10.1111/eva.12694)

Table S2. Relation of the 20 most recent articles on zooplankton metabarcoding or metagenetics. See text and Fig. S1 for further details.

1. Berry, T. E., Coghlan, M. L., Saunders, B. J., Richardson, A. J., Power, M., Harvey, E., Jarman, S., Berry, O., *et al.* (2023) A 3-year plankton DNA metabarcoding survey reveals marine biodiversity patterns in Australian coastal waters. *Diversity and Distributions*, **29**, 862–878. [10.1111/ddi.13699](https://doi.org/10.1111/ddi.13699).
2. Descôteaux, R., Huserbråten, M., Jørgensen, L., Renaud, P., Ingvaldsen, R., Ershova, E., and Bluhm, B. (2022) Origin of marine invertebrate larvae on an Arctic inflow shelf. *Mar Ecol Prog Ser*, **699**, 1–17.
3. Dischereit, A., Wangensteen, O. S., Præbel, K., Auel, H., and Havermans, C. (2022) Using DNA Metabarcoding to Characterize the Prey Spectrum of Two Co-Occurring *Themisto* Amphipods in the Rapidly Changing Atlantic-Arctic Gateway Fram Strait. *Genes*, **13** [10.3390/genes13112035](https://doi.org/10.3390/genes13112035).
4. Ershova, E. A., Wangensteen, O. S., and Falkenhaug, T. (2023) Mock samples resolve biases in diversity estimates and quantitative interpretation of zooplankton metabarcoding data. *Marine Biodiversity*, **53**, 66. [10.1007/s12526-023-01372-x](https://doi.org/10.1007/s12526-023-01372-x).
5. Feng, Y., Sun, D., Shao, Q., Fang, C., and Wang, C. (2022) Mesozooplankton biodiversity, vertical assemblages, and diel migration in the western tropical Pacific Ocean revealed by eDNA metabarcoding and morphological methods. *Frontiers in Marine Science*, **9**.
6. Feng, Y., Sun, D., Shao, Q., Fang, C., and Wang, C. (2023) COI metabarcoding better reveals the seasonal variations in the zooplankton community in the western Pacific Warm Pool. *Ecological Indicators*, **156**, 111183. [10.1016/j.ecolind.2023.111183](https://doi.org/10.1016/j.ecolind.2023.111183).
7. Gasca-Pineda, J., Galindo-Sánchez, C. E., Martinez-Mercado, M. A., Jiménez-Rosenberg, S. P. A., Hereu, C. M., Nakamura, Y., Herzka, S. Z., Compaire, J. C., *et al.* (2023) Community structure and diversity of five groups of zooplankton in the Perdido region of the Gulf of Mexico using DNA metabarcoding. *Aquatic Ecology*, **57**, 149–164. [10.1007/s10452-022-10002-w](https://doi.org/10.1007/s10452-022-10002-w).
8. Govender, A., Groeneveld, J. C., Singh, S. P., and Willows-Munro, S. (2022a) Metabarcoding of zooplankton confirms southwards dispersal of decapod crustacean species in the western Indian Ocean. *African Journal of Marine Science*, **44**, 279–289. [10.2989/1814232X.2022.2108144](https://doi.org/10.2989/1814232X.2022.2108144).
9. Govender, Ashrenee, Singh, S., Groeneveld, J., Pillay, S., and Willows-Munro, S. (2022b) Experimental validation of taxon-specific mini-barcode primers for metabarcoding of zooplankton. *Ecological Applications*, **32**, e02469. [10.1002/eap.2469](https://doi.org/10.1002/eap.2469).
10. Guy-Haim, T., Dubinsky-Velasquez, X., Terbiyik Kurt, T., Di Capua, I., Mazzocchi, M. G., and Morov, A. (2022) A new record of the rapidly spreading calanoid copepod *Pseudodiaptomus marinus* (Sato, 1913) in the Levantine Sea using multi-marker metabarcoding. *BioInvasions Records*, **11**, 1–13.<https://doi.org/10.3391/bir.2022.11.4.14>.
11. Matthews, S. A. and Blanco-Bercial, L. (2023) Divergent patterns of zooplankton connectivity in the epipelagic and mesopelagic zones of the eastern North Pacific. *Ecology and Evolution*, **13**, e10664. [10.1002/ece3.10664](https://doi.org/10.1002/ece3.10664).
12. Matthews, S. A. and Ohman, M. D. (2023) A space-for-time framework for forecasting the effects of ocean stratification on zooplankton vertical habitat use and trait composition. *Limnology and Oceanography*, **68**, 2688–2702. [10.1002/lno.12450](https://doi.org/10.1002/lno.12450).
13. Min, M. A., Needham, D. M., Sudek, S., Truelove, N. K., Pitz, K. J., Chavez, G. M., Poirier, C., Gardeler, B., *et al.* (2023) Ecological divergence of a mesocosm in an eastern boundary upwelling system assessed with multi-marker environmental DNA metabarcoding. *Biogeosciences*, **20**, 1277–1298. [10.5194/bg-20-1277-2023](https://doi.org/10.5194/bg-20-1277-2023).
14. Ohnesorge, A., John, U., Taudien, S., Neuhaus, S., Kuczynski, L., and Laakmann, S. (2023) Capturing drifting species and molecules—Lessons learned from integrated approaches to assess marine metazoan diversity in highly dynamic waters. *Environmental DNA*, **5**, 1541–1556. [10.1002/edn3.478](https://doi.org/10.1002/edn3.478).
15. O’Rorke, R., van der Reis, A., von Ammon, U., Beckley, L. E., Pochon, X., Zaiko, A., and Jeffs, A. (2022) eDNA metabarcoding shows latitudinal eukaryote micro- and mesoplankton diversity stabilizes across oligotrophic region of a >3000 km longitudinal transect in the Indian Ocean. *Deep Sea Research Part II: Topical Studies in Oceanography*, **205**, 105178. [10.1016/j.dsr2.2022.105178](https://doi.org/10.1016/j.dsr2.2022.105178).
16. Qian, B., Miao, X., and Xu, F. (2023) Temporal dynamics of zooplankton community in an oyster farming area of the Yellow Sea in China via metabarcoding. *Frontiers in Marine Science*, **10**.
17. Qihang, L., Yuanming, C., Qianwen, S., Zaiming, W., Wei, X., Yadong, Z., Dong, S., Xiaohui, X., *et al.* (2022) Metabarcoding survey of meroplankton communities in the South China Sea and Philippine Sea: Shedding light on inter-basin biogeography in the West Pacific. *Frontiers in Marine Science*, **9**.
18. Schroeder, A., Camatti, E., Pansera, M., and Pallavicini, A. (2023) Feeding pressure on meroplankton by the invasive ctenophore Mnemiopsis leidyi. *Biological Invasions*, **25**, 2007–2021. [10.1007/s10530-023-03023-5](https://doi.org/10.1007/s10530-023-03023-5).
19. Sun, Y., Li, H., Wang, X., Jin, Y., Nagai, S., and Lin, S. (2023) Phytoplankton and Microzooplankton Community Structure and Assembly Mechanisms in Northwestern Pacific Ocean Estuaries with Environmental Heterogeneity and Geographic Segregation. *Microbiol Spectr*, **11**, e04926-22.<https://doi.org/10.1128/spectrum.04926-22>.
20. Yebra, L., García-Gómez, C., Valcárcel-Pérez, N., Hernández de Rojas, A., Blanco-Bercial, L., Castro, M. C., Gómez-Jakobsen, F., and Mercado, J. M. (2022) Assessment of short-term spatio-temporal variability in the structure of mesozooplankton communities integrating microscopy and multigene high-throughput sequencing. *Estuarine, Coastal and Shelf Science*, **276**, 108038. [10.1016/j.ecss.2022.108038](https://doi.org/10.1016/j.ecss.2022.108038).

Table S3. Relation of the 30 articles in which miniCOI performance was reported together with microscopy and/or another universal marker. See text and Fig. S1 for further details.

1. Bucklin, A., Batta-Lona, P. G., Questel, J. M., Wiebe, P. H., Richardson, D. E., Copley, N. J., and O’Brien, T. D. (2022) COI Metabarcoding of Zooplankton Species Diversity for Time-Series Monitoring of the NW Atlantic Continental Shelf. *Frontiers in Marine Science*, **9**.
2. Carroll, E. L., Gallego, R., Sewell, M. A., Zeldis, J., Ranjard, L., Ross, H. A., Tooman, L. K., O’Rorke, R., *et al.* (2019) Multi-locus DNA metabarcoding of zooplankton communities and scat reveal trophic interactions of a generalist predator. *Scientific Reports*, **9**, 281. [10.1038/s41598-018-36478-x](https://doi.org/10.1038/s41598-018-36478-x).
3. Cicala, F., Arteaga, M. C., Herzka, S. Z., Hereu, C. M., Jimenez-Rosenberg, S. P. A., Saavedra-Flores, A., Robles-Flores, J., Gomez, R., *et al.* (2022) Environmental conditions drive zooplankton community structure in the epipelagic oceanic water of the southern Gulf of Mexico: A molecular approach. *Molecular Ecology*, **31**, 546–561. [10.1111/mec.16251](https://doi.org/10.1111/mec.16251).
4. Clarke, L. J., Beard, J. M., Swadling, K. M., and Deagle, B. E. (2017) Effect of marker choice and thermal cycling protocol on zooplankton DNA metabarcoding studies. *Ecology and Evolution*, **7**, 873–883. [10.1002/ece3.2667](https://doi.org/10.1002/ece3.2667).
5. Clarke, L. J., Trebilco, R., Walters, A., Polanowski, A. M., and Deagle, B. E. (2020) DNA-based diet analysis of mesopelagic fish from the southern Kerguelen Axis. *Deep Sea Research Part II: Topical Studies in Oceanography*, **174** [10.1016/j.dsr2.2018.09.001](https://doi.org/10.1016/j.dsr2.2018.09.001).
6. Coguiec, E., Ershova, E. A., Daase, M., Vonnahme, T. R., Wangensteen, O. S., Gradinger, R., Præbel, K., and Berge, J. (2021) Seasonal Variability in the Zooplankton Community Structure in a Sub-Arctic Fjord as Revealed by Morphological and Molecular Approaches. *Frontiers in Marine Science*, **8**.
7. Deagle, B. E., Clarke, L. J., Kitchener, J. A., Polanowski, A. M., and Davidson, A. T. (2018) Genetic monitoring of open ocean biodiversity: An evaluation of DNA metabarcoding for processing continuous plankton recorder samples. *Molecular Ecology Resources*, **18**, 391–406. [10.1111/1755-0998.12740](https://doi.org/10.1111/1755-0998.12740).
8. Djurhuus, A., Pitz, K., Sawaya, N. A., Rojas-Márquez, J., Michaud, B., Montes, E., Muller-Karger, F., and Breitbart, M. (2018) Evaluation of marine zooplankton community structure through environmental DNA metabarcoding. *Limnology and Oceanography: Methods*, **16**, 209–221. [10.1002/lom3.10237](https://doi.org/10.1002/lom3.10237).
9. Ershova, E. A., Wangensteen, O. S., Descoteaux, R., Barth-Jensen, C., and Præbel, K. (2021) Metabarcoding as a quantitative tool for estimating biodiversity and relative biomass of marine zooplankton. *ICES Journal of Marine Science*, **78**, 3342–3355. [10.1093/icesjms/fsab171](https://doi.org/10.1093/icesjms/fsab171).
10. Ershova, E. A., Wangensteen, O. S., and Falkenhaug, T. (2023) Mock samples resolve biases in diversity estimates and quantitative interpretation of zooplankton metabarcoding data. *Marine Biodiversity*, **53**, 66. [10.1007/s12526-023-01372-x](https://doi.org/10.1007/s12526-023-01372-x).
11. Feng, Y., Sun, D., Shao, Q., Fang, C., and Wang, C. (2022) Mesozooplankton biodiversity, vertical assemblages, and diel migration in the western tropical Pacific Ocean revealed by eDNA metabarcoding and morphological methods. *Frontiers in Marine Science*, **9**.
12. Feng, Y., Sun, D., Shao, Q., Fang, C., and Wang, C. (2023) COI metabarcoding better reveals the seasonal variations in the zooplankton community in the western Pacific Warm Pool. *Ecological Indicators*, **156**, 111183. [10.1016/j.ecolind.2023.111183](https://doi.org/10.1016/j.ecolind.2023.111183).
13. Guy-Haim, T., Dubinsky-Velasquez, X., Terbiyik Kurt, T., Di Capua, I., Mazzocchi, M. G., and Morov, A. (2022) A new record of the rapidly spreading calanoid copepod *Pseudodiaptomus marinus* (Sato, 1913) in the Levantine Sea using multi-marker metabarcoding. *BioInvasions Records*, **11**, 1–13.<https://doi.org/10.3391/bir.2022.11.4.14>.
14. Harvey, J. B. J., Johnson, S. B., Fisher, J. L., Peterson, W. T., and Vrijenhoek, R. C. (2017) Comparison of morphological and next generation DNA sequencing methods for assessing zooplankton assemblages. *Journal of Experimental Marine Biology and Ecology*, **487**, 113–126. [10.1016/j.jembe.2016.12.002](https://doi.org/10.1016/j.jembe.2016.12.002).
15. Harvey, J., Fisher, J., Ryan, J., Johnson, S., Peterson, W., and Vrijenhoek, R. (2018) Changes in zooplankton assemblages in northern Monterey Bay, California, during a fall transition. *Mar Ecol Prog Ser*, **604**, 99–120.
16. Machida, R. J., Kurihara, H., Nakajima, R., Sakamaki, T., Lin, Y.-Y., and Furusawa, K. (2021) Comparative analysis of zooplankton diversities and compositions estimated from complement DNA and genomic DNA amplicons, metatranscriptomics, and morphological identifications. *ICES Journal of Marine Science*, **78**, 3428–3443. [10.1093/icesjms/fsab084](https://doi.org/10.1093/icesjms/fsab084).
17. Matthews, S. A., Goetze, E., and Ohman, M. D. (2021) Recommendations for interpreting zooplankton metabarcoding and integrating molecular methods with morphological analyses. *ICES Journal of Marine Science*, **78**, 3387–3396. [10.1093/icesjms/fsab107](https://doi.org/10.1093/icesjms/fsab107).
18. Matthews, S. A. and Blanco-Bercial, L. (2023) Divergent patterns of zooplankton connectivity in the epipelagic and mesopelagic zones of the eastern North Pacific. *Ecology and Evolution*, **13**, e10664. [10.1002/ece3.10664](https://doi.org/10.1002/ece3.10664).
19. Matthews, S. A. and Ohman, M. D. (2023) A space-for-time framework for forecasting the effects of ocean stratification on zooplankton vertical habitat use and trait composition. *Limnology and Oceanography*, **68**, 2688–2702. [10.1002/lno.12450](https://doi.org/10.1002/lno.12450).
20. Novotny, A., Jan, K. M. G., Dierking, J., and Winder, M. (2022) Niche partitioning between planktivorous fish in the pelagic Baltic Sea assessed by DNA metabarcoding, qPCR and microscopy. *Scientific Reports*, **12**, 10952. [10.1038/s41598-022-15116-7](https://doi.org/10.1038/s41598-022-15116-7).
21. Ohnesorge, A., John, U., Taudien, S., Neuhaus, S., Kuczynski, L., and Laakmann, S. (2023) Capturing drifting species and molecules—Lessons learned from integrated approaches to assess marine metazoan diversity in highly dynamic waters. *Environmental DNA*, **5**, 1541–1556. [10.1002/edn3.478](https://doi.org/10.1002/edn3.478).
22. Pappalardo, P., Collins, A. G., Pagenkopp Lohan, K. M., Hanson, K. M., Truskey, S. B., Jaeckle, W., Ames, C. L., Goodheart, J. A., *et al.* (2021) The role of taxonomic expertise in interpretation of metabarcoding studies. *ICES Journal of Marine Science*, **78**, 3397–3410. [10.1093/icesjms/fsab082](https://doi.org/10.1093/icesjms/fsab082).
23. Pitz, K. J., Guo, J., Johnson, S. B., Campbell, T. L., Zhang, H., Vrijenhoek, R. C., Chavez, F. P., and Geller, J. (2020) Zooplankton biogeographic boundaries in the California Current System as determined from metabarcoding. *PLOS ONE*, **15**, e0235159. [10.1371/journal.pone.0235159](https://doi.org/10.1371/journal.pone.0235159).
24. Questel, J. M., Hopcroft, R. R., DeHart, H. M., Smoot, C. A., Kosobokova, K. N., and Bucklin, A. (2021) Metabarcoding of zooplankton diversity within the Chukchi Borderland, Arctic Ocean: improved resolution from multi-gene markers and region-specific DNA databases. *Marine Biodiversity*, **51**, 4. [10.1007/s12526-020-01136-x](https://doi.org/10.1007/s12526-020-01136-x).
25. Schroeder, A., Stanković, D., Pallavicini, A., Gionechetti, F., Pansera, M., and Camatti, E. (2020) DNA metabarcoding and morphological analysis - Assessment of zooplankton biodiversity in transitional waters. *Marine Environmental Research*, **160**, 104946. [10.1016/j.marenvres.2020.104946](https://doi.org/10.1016/j.marenvres.2020.104946).
26. Singh, S., Groeneveld, J., Huggett, J., Naidoo, D., Cedras, R., and Willows-Munro, S. (2021) Metabarcoding of marine zooplankton in South Africa. *African Journal of Marine Science*, **43**, 147–159. [10.2989/1814232X.2021.1919759](https://doi.org/10.2989/1814232X.2021.1919759).
27. Stefanni, S., Stanković, D., Borme, D., de Olazabal, A., Juretić, T., Pallavicini, A., and Tirelli, V. (2018) Multi-marker metabarcoding approach to study mesozooplankton at basin scale. *Scientific Reports*, **8**, 12085. [10.1038/s41598-018-30157-7](https://doi.org/10.1038/s41598-018-30157-7).
28. Suter, L., Polanowski, A. M., Clarke, L. J., Kitchener, J. A., and Deagle, B. E. (2021) Capturing open ocean biodiversity: Comparing environmental DNA metabarcoding to the continuous plankton recorder. *Molecular Ecology*, **30**, 3140–3157. [10.1111/mec.15587](https://doi.org/10.1111/mec.15587).
29. Yebra, L., García-Gómez, C., Valcárcel-Pérez, N., Hernández de Rojas, A., Blanco-Bercial, L., Castro, M. C., Gómez-Jakobsen, F., and Mercado, J. M. (2022) Assessment of short-term spatio-temporal variability in the structure of mesozooplankton communities integrating microscopy and multigene high-throughput sequencing. *Estuarine, Coastal and Shelf Science*, **276**, 108038. [10.1016/j.ecss.2022.108038](https://doi.org/10.1016/j.ecss.2022.108038).
30. Zhang, G. K., Chain, F. J. J., Abbott, C. L., and Cristescu, M. E. (2018) Metabarcoding using multiplexed markers increases species detection in complex zooplankton communities. *Evolutionary Applications*, **11**, 1901–1914. [10.1111/eva.12694](https://doi.org/10.1111/eva.12694)

Table S4. This table is an Excel file.

Table S4. Review of miniCOI metabarcoding performance with marine zooplankton taxa. Detailed data on Appendicularians and *Oithona similis* and other locally abundant taxa that were quoted as showing pronounced bias or a detection failure in the reviewed papers (see quotes and notes in the two columns about performance). Articles were ranked by 1) miniCOI primer combination, from those with less ambiguities in the forward primer to those more degenerated (i.e. Leray XT, see Table I) and, finally, those applying less common primers or a cocktail of primers for the miniCOI region; 2) Publication year from most recent to oldest, within each primer combination section. First column indicates reference number in Table S3. Columns 2-3 indicate whether miniCOI performance was compared either with microscopy and or with an alternative universal marker. Methodological details include (in this order): 1. Sample type (sampling device), 2. Geographical location of samples, 3. Sequencing device, 4. Reference database/s for the COI region, and, 5. Alternative universal marker (if any). References to Figures and Tables refer to the original articles. eDNAss: environmental DNA *sensu stricto* (from filtered water, not from community net sample). Fail: no detection (false negative), Bias: pronounced bias (detected but at notably lower levels: more than an order of magnitude below in relative frequency that when compared to microscopy/alternative marker), N/A: either non field/mock presence or non reported data, OK: rest of situations (= from fair to good correspondence with microscopy/alternative marker).

Table S5*.* Mismatches in *Oithona similis* for miniCOI forward primer. Location of mismatches in the miniCOI forward primer region for *Oithona similis*, obtained from 247 sequences found in MZGdb; ambiguous nucleotides (W=A/T; Y=C/T; R=A/G) and inosines (I, considered as universal nucleotide, see text for further details) in bold. Number of sequences per nucleotide position in blue when fails with just Leray´s mlCOIintF forward primer; red when both mlCOIintF and Leray XT forward primers fail.

| Nucleotide position (5´to 3´) | #1 | #2 | #3 | #4 | #5 | #6 | #7 | #8 | #9 | #10 | #11 | #12 | #13 | #14 | #15 | #16 | #17 | #18 | #19 | #20 | #21 | #22 | #23 | #24 | #25 | #26 |
| --- | --- | --- | --- | --- | --- | --- | --- | --- | --- | --- | --- | --- | --- | --- | --- | --- | --- | --- | --- | --- | --- | --- | --- | --- | --- | --- |
| Leray mlCOIintF forward primer | G | G | **W** | A | C | **W** | G | G | **W** | T | G | A | A | C | **W** | G | T | **W** | T | A | **Y** | C | C | **Y** | C | C |
| Leray XT forward primer | G | G | **W** | A | C | **W** | **R** | G | **W** | T | G | **R** | A | C | **W** | **I** | T | **I** | T | A | **Y** | C | C | **Y** | C | C |
| *Oithona similis* MZG 247 sequences | * | * | C/G/T | * | * | A/C/G/T | *(G) | * | C/T | * | * | A/G | * | * | A/G/T | *(G) | * | A/G/T | * | * | C/T | * | * | A/C/G/T | * | * |
| Number of sequences per nucleotide |  |  | 49/5/193 |  |  | 155/76/10/6 |  |  | 5/242 |  |  | 123/124 |  |  | 25/1/221 |  |  | 2/224/21 |  |  | 197/50 |  |  | 39/148/4/76 |  |  |
| % Match mlCOIintF | 100 | 100 | 78 | 100 | 100 | 65 | 100 | 100 | 98 | 100 | 100 | 50 | 100 | 100 | 100 | 100 | 100 | 9 | 100 | 100 | 100 | 100 | 100 | 83 | 100 | 100 |
| % Match LerayXT | 100 | 100 | 78 | 100 | 100 | 65 | 100 | 100 | 98 | 100 | 100 | 100 | 100 | 100 | 100 | 100 | 100 | 100 | 100 | 100 | 100 | 100 | 100 | 83 | 100 | 100 |

Table S6. Mismatches in *Oithona nana* for miniCOI forward primer. Location of mismatches in the miniCOI forward primer region for *Oithona nana*, obtained from 16 sequences found in MZGdb; ambiguous nucleotides in bold. Number of sequences per nucleotide position in blue when fails with just Leray´s mlCOIintF forward primer; red when both mlCOIintF and Leray XT forward primers fail. Two haplotypes (A and B) were reported for this species.

| Nucleotide position (5´to 3´) | | #1 | #2 | #3 | #4 | #5 | #6 | #7 | #8 | #9 | #10 | #11 | #12 | #13 | #14 | #15 | #16 | #17 | #18 | #19 | #20 | #21 | #22 | #23 | #24 | #25 | #26 |
| --- | --- | --- | --- | --- | --- | --- | --- | --- | --- | --- | --- | --- | --- | --- | --- | --- | --- | --- | --- | --- | --- | --- | --- | --- | --- | --- | --- |
| Leray mlCOIintF forward primer | | G | G | **W** | A | C | **W** | G | G | **W** | T | G | A | A | C | **W** | G | T | **W** | T | A | **Y** | C | C | **Y** | C | C |
| Leray XT forward primer | | G | G | **W** | A | C | **W** | **R** | G | **W** | T | G | **R** | A | C | **W** | **I** | T | **I** | T | A | **Y** | C | C | **Y** | C | C |
| *Oithona nana* MZG 16 sequences | | * | * | *(G) | * | * |  | *(G) | * |  | * | * | *(A) | * | * |  | *(G) | * |  | * | * |  | * | * |  | * | * |
| A: KU982947-56, 10 sequences | |  |  |  |  |  | T |  |  | T |  |  |  |  |  | A |  |  | G |  |  | C |  |  | C |  |  |
| B: OM765165-70, 6 sequences | |  |  |  |  |  | C |  |  | G |  |  |  |  |  | C |  |  | T |  |  | T |  |  | G |  |  |
| Number of sequences per nucleotide | |  |  | 16 |  |  | 6/10 |  |  | 6/10 |  |  |  |  |  | 10/6 |  |  | 10/6 |  |  | 10/6 |  |  | 10/6 |  |  |
| % Match mlCOIintF | *O. nana* A | 100 | 100 | 0 | 100 | 100 | 100 | 100 | 100 | 100 | 100 | 100 | 100 | 100 | 100 | 100 | 100 | 100 | 0 | 100 | 100 | 100 | 100 | 100 | 100 | 100 | 100 |
|  | *O. nana* B | 100 | 100 | 0 | 100 | 100 | 0 | 100 | 100 | 0 | 100 | 100 | 100 | 100 | 100 | 0 | 100 | 100 | 100 | 100 | 100 | 100 | 100 | 100 | 0 | 100 | 100 |
| % Match LerayXT | *O. nana* A | 100 | 100 | 0 | 100 | 100 | 100 | 100 | 100 | 100 | 100 | 100 | 100 | 100 | 100 | 100 | 100 | 100 | 100 | 100 | 100 | 100 | 100 | 100 | 100 | 100 | 100 |
|  | *O. nana* B | 100 | 100 | 0 | 100 | 100 | 0 | 100 | 100 | 0 | 100 | 100 | 100 | 100 | 100 | 0 | 100 | 100 | 100 | 100 | 100 | 100 | 100 | 100 | 0 | 100 | 100 |

Table S7. Mismatches in *Microsetella norvegica* for miniCOI forward primer. Location of mismatches in the miniCOI forward primer region for *Microsetella norvegica*, obtained from 24 sequences found in MZGdb; ambiguous nucleotides in bold. Number of sequences per nucleotide position in blue when fails with just Leray´s mlCOIintF forward primer; red when both mlCOIintF and Leray XT forward primers fail.

| Nucleotide position (5´to 3´) | #1 | #2 | #3 | #4 | #5 | #6 | #7 | #8 | #9 | #10 | #11 | #12 | #13 | #14 | #15 | #16 | #17 | #18 | #19 | #20 | #21 | #22 | #23 | #24 | #25 | #26 |
| --- | --- | --- | --- | --- | --- | --- | --- | --- | --- | --- | --- | --- | --- | --- | --- | --- | --- | --- | --- | --- | --- | --- | --- | --- | --- | --- |
| Leray mlCOIintF forward primer | G | G | **W** | A | C | **W** | G | G | **W** | T | G | A | A | C | **W** | G | T | **W** | T | A | **Y** | C | C | **Y** | C | C |
| Leray XT forward primer | G | G | **W** | A | C | **W** | **R** | G | **W** | T | G | **R** | A | C | **W** | **I** | T | **I** | T | A | **Y** | C | C | **Y** | C | C |
| *Microsetella norvegica* MZG 24 sequences | * | * | A/C/G | * | * | A/T | *(G) | * | C/G | * | * | *(A) | * | * | A/C/G | *(G) | * | A/G/T | * | * | *(C) | * | * | C/G/T | * | * |
| Number of sequences per nucleotide |  |  | 3/2/19 |  |  | 22/2 |  |  | 5/19 |  |  |  |  |  | 2/19/3 |  |  | 3/19/2 |  |  |  |  |  | 3/19/2 |  |  |
| % Match mlCOIintF | 100 | 100 | 13 | 100 | 100 | 100 | 100 | 100 | 0 | 100 | 100 | 100 | 100 | 100 | 8 | 100 | 100 | 21 | 100 | 100 | 100 | 100 | 100 | 21 | 100 | 100 |
| % Match LerayXT | 100 | 100 | 13 | 100 | 100 | 100 | 100 | 100 | 0 | 100 | 100 | 100 | 100 | 100 | 8 | 100 | 100 | 100 | 100 | 100 | 100 | 100 | 100 | 21 | 100 | 100 |

Table S8. List of primers used for amplification and sequencing of appendicularian COI in this study.

| Direction | Primer | Sequence | Reference |
| --- | --- | --- | --- |
| Forward | LCO1490 | GGTCAACAAATCATAAAGATATTGG | Folmer et al., 1994 |
| Reverse | HCO2198 | TAAACTTCAGGGTGACCAAAAAATCA | Folmer et al., 1994 |
| Reverse | coi-930r | GCAGTAAAATAAGCACGAGAATC | Garić and Batistić, 2016 |
| Forward | odlco | GTTCGGTAAATCATAAAGACATTGG | this study |
| Forward | longcoif | TGATGGGTGTTCCTGATATGG | this study |
| Reverse | ofus700r | CAAAGAACCAAAATAGATGCTGGAA | this study |
| Reverse | longcoir | GAAAGAAGTATTAAAATGACGATC | this study |
| Reverse | kochco | TAAACTTCTGGATGCCTAAAAAATCA | this study |
| Reverse | od1440nr | CATGATACTTACTATGTTGTTGCTCA | this study |

Table S9. Appendicularian COI sequences obtained in this study and from GenBank used for miniCOI primer mismatch analysis in Table IV.

| Sequences obtained in this study* | | | | | | |
| --- | --- | --- | --- | --- | --- | --- |
| Acc. number | Species | | Forward primer | Reverse primer | | Sampling location |
| PP339655 | *Megalocercus abyssorum* | | odlco | ofus700r | | Croatia |
| PP339656 | *Oikopleura longicauda* | | longcoif | longcoir | | Croatia |
| PP339657 | *Oikopleura longicauda* | | longcoif | longcoir | | Croatia |
| PP339658 | *Oikopleura longicauda* | | longcoif | longcoir | | Croatia |
| PP339659 | *Oikopleura fusiformis* | | LCO1490 | coi-930r | | Croatia |
| PP339660 | *Oikopleura dioica* | | LCO1490 | kochco | | Norway |
| PP339661 | *Oikopleura albicans* | | LCO1490 | kochco | | Croatia |
| PP339662 | *Stegosoma magnum* | | LCO1490 | kochco | | Croatia |
| PP339663 | *Appendicularia sicula* | | LCO1490 | kochco | | Croatia |
| PP339664 | *Fritillaria borealis sargassi* | | odlco | coi-930r | | Croatia |
| PP339665 | *Fritillaria pellucida* | | LCO1490 | coi-930r | | Croatia |
| PP339666 | *Fritillaria formica tuberculata* | | LCO1490 | kochco | | Croatia |
| PP339667 | *Kowalevskia tenuis* | | LCO1490 | HCO2198 | | Croatia |
| PP339668 | *Kowalevskia oceanica* | | LCO1490 | coi-930r | | Croatia |
| PP339669 | *Kowalevskia oceanica* | | LCO1490 | od1440nr | | Croatia |
| Sequences obtained from GenBank | | | | | | |
| Acc. number | Species | Reference | | | Sampling location | |
| SCLE01415711 | *Bathochordaeus stygius* | Naville et al., 2019 | | | USA, Monterey Bay | |
| SCLF01725989 | *Mesochordaeus erythrocephalus* | Naville et al., 2019 | | | USA, Monterey Bay | |
| LC222754 | *Oikopleura longicauda* | Sakaguchi et al., 2017 | | | Japan | |
| SCLD01101138 | *Oikopleura longicauda* | Naville et al., 2019 | | | USA, La Jolla Shores | |
| GCJN01047493 | *Oikopleura dioica* | Wang et al., 2015 | | | Japan | |
| SAMN00177767 | *Oikopleura dioica* | Denoeud et al., 2010 | | | Norway | |

*Appendicularian DNA and RNA isolation, RNA reverse transcription and PCR: Samples were collected using 53-μm Nansen zooplankton net equipped with the closing mechanism. Upon collection the collected zooplankton was filtered through 53-μm mesh to remove seawater and placed in 94% ethanol for sorting. The individual animals were separated and placed in separate vials in 100% acetone. Within 24 hours the individual animals were dried at 55°C for half an hour and placed into lysis solution (Garić and Batistić, 2022) for 1 hour which was the first step of both DNA and RNA isolation procedure. DNA was purified from the lysate following the ammonium-acetate based protocol described in Garić and Batistić (2022). RNA was purified using the Trizol method: 50 μl of lysate was mixed with 500 μl of Trizol and well mixed; after 5 min i 100 μl of chloroform was added and mixed well; the mixture was centrifuged for 15 min at 12000 g; top layer was transferred into new tubes; 250 μl of isopropanol is added, well mixed and left to set for 10 min; the mixture was centrifuged for 10 min at 12000 g; the supernatant is removed and the RNA pellet is washed with 1 ml of 75% ethanol; after the washing step the tubes were centrifuged for 10 min at 12000 g; supernatant removed and RNA pellet air dried; after drying, RNA was dissolved in water. First strand synthesis was done with ProtoScript® II Reverse Transcriptase (New England Biolabs) following the manufacturer instructions and using poly-T primer 23 nucleotides long. PCR was performed using the obtained ssDNA in a 50 μl PCR mix containing: 1 × PCR buffer, 0.2 mM of each dNTP, 3 mM, MgCl2, 0.2 μM of each primer, 1.2 U of recombinant Taq polymerase (Thermo Scientific) and 1 μl of template DNA. All amplifications were performed using a PCR programme with a 2 min denaturation step at 94°C, with 40 subsequent cycles of 94°C for 20 s, 45°C for 1 min, 72°C for 1 min, and a final extension step at 72°C for 5 min. Different primer pairs were tested until successful amplification was obtained (Table S8).

Table S10. Mismatches in Class Appendicularia for the miniCOI reverse primer. MiniCOI reverse primer jgHCO2198 (Geller et al., 2013) mismatch analysis in Appendicularia at the non-conserved position #17 (where a cytosine, “C”, is present in primer jgHCO2198). Light grey indicates mismatch with primer. Appendicularian species containing poly-T inserts in their mitochondrial DNA shown in black.

| Acc. number | Species | #17 | Mismatches | poly-Ts |
| --- | --- | --- | --- | --- |
| SCLE01415711 | *Bathochordaeus stygius* |  | 0 |  |
| SCLF01725989 | *Mesochordaeus erythrocephalus* | T | 1 |  |
| LC222754 | *Oikopleura longicauda* |  | 0 |  |
| SCLD01101138 | *Oikopleura longicauda* |  | 0 |  |
| PP339659 | *Oikopleura fusiformis* |  | 0 |  |
| SAMN00177767 | *Oikopleura dioica* |  | 0 |  |
| GCJN01047493 | *Oikopleura dioica* |  | 0 |  |
| PP339664 | *Fritillaria borealis sargassi* | T | 1 |  |
| PP339665 | *Fritillaria pellucida* | T | 1 |  |
| PP339668 | *Kowalevskia oceanica* | T | 1 |  |
| PP339669 | *Kowalevskia oceanica* | T | 1 |  |

Table S11. Thaliacean sequences from GenBank used for mini-COI primer mismatch analysis.

| Acc. number | Species | Reference | Sampling location |
| --- | --- | --- | --- |
| OP437494 | *Dolioletta advena* | Garić and Batistić, 2022 | Croatia |
| OP437493 | *Dolioletta advena* |  |  |
| OP437492 | *Dolioletta advena* |  |  |
| OP437491 | *Dolioletta gegenbauri* |  |  |
| OP437490 | *Doliolina krohni* |  |  |
| OP437489 | *Doliolina muelleri* |  |  |
| OP437495 | *Doliolum nationalis* |  |  |
| AB176541 | *Doliolum nationalis* | Yokobori et al., 2005 | Japan |
| OP437487 | *Doliolum denticulatum* | Garić and Batistić, 2022 | Croatia |
| OP437488 | *Pyrosoma atlanticum* |  |  |
| MT998285 | *Thalia longicauda* | Peter et al., unpublished | unknown |
| MH626415 | *Thalia democratica* | Peter et al., unpublished | unknown |
| KT818686 | *Brooksia lacromae* | Garić and Batisić, 2016 | Croatia |
| LC333181 | *Salpa fusiformis* | Goodall-Copestake, 2018 | Atlantic Ocean, near Gough Island |
| LC333180 | *Salpa thompsoni* | Goodall-Copestake, 2018 | Southern Ocean, near Elephant Island |

Table S12. Mismatches in Class Thaliacea for the miniCOI reverse primer. Mismatches of the reverse primer jgHCO2198 (Geller et al., 2013) in Thaliacea at the non-conserved position #17 (where a cytosine, “C”, is present in primer jgHCO2198). Light grey letters represent mismatches with primer.

| Acc. number | Species | #17 | Mismatches |
| --- | --- | --- | --- |
| OP437494 | *Dolioletta advena* | T | 1 |
| OP437493 | *Dolioletta advena* | T | 1 |
| OP437492 | *Dolioletta advena* | T | 1 |
| OP437491 | *Dolioletta gegenbauri* | T | 1 |
| OP437490 | *Doliolina krohni* | T | 1 |
| OP437489 | *Doliolina muelleri* | T | 1 |
| AB176541 | *Doliolum nationalis* | T | 1 |
| OP437487 | *Doliolum denticulatum* | T | 1 |
| OP437488 | *Pyrosoma atlanticum* | T | 1 |
| KT818686 | *Brooksia lacromae* | T | 1 |
| LC333181 | *Salpa fusiformis* |  | 0 |
| LC333180 | *Salpa thompsoni* |  | 0 |
